# Nutritional optimization for bioprocess production of staphyloxanthin from *Staphylococcus aureus* with response surface methodology: promising anticancer scaffold targeting EGFR inhibition

**DOI:** 10.1186/s12934-025-02717-w

**Published:** 2025-05-06

**Authors:** Ahmed M. Nosair, Ahmed A. Abdelaziz, Amal M. Abo-Kamer, Lamiaa A. Al-Madboly, Mahmoud H. Farghali

**Affiliations:** Department of Microbiology and Immunology, Faculty of Pharmacy, Tanta University, Tanta, Egypt

**Keywords:** Staphyloxanthin, RSM optimization, NSC lung cancer, Apoptosis, EGFR

## Abstract

**Background:**

Staphyloxanthin (STX) is a secondary metabolite pigment associated with membrane structures, recognized for its significant antioxidant properties. It plays a crucial role in combating reactive oxygen species (ROS), positioning it as a promising and effective alternative in cancer treatment. This study focused on enhancing the production of STX pigment by employing statistical optimization of media components, alongside the evaluation of its safety and anticancer properties.

**Results:**

A total of 59 *Staphylococcus aureus* isolates were screened and quantitatively estimated for STX production. The best pigment-producing isolate was identified based on molecular phylogenetic analysis as *S. aureus* A2, with accession number PP197164. A Box-Wilson central composite design was employed to evaluate the intricate interactions among six variables affecting the pigment yield. The most optimal conditions resulted in the highest production of STX of OD_456_ = 0.328, which is approximately 1.5-fold greater than the yield (OD_456_ = 0.215) obtained from OFAT optimization. The final response surface model fitting the data achieved a R² of 0.8748. STX exhibited marked cytotoxicity against the A549 NSCLC cell line with IC50 of 57.3 µg/mL, a safe dose in normal Vero cells. The anticancer activity of STX was predominantly mediated by the apoptotic pathway, as confirmed by confocal microscopy, the annexin V-FITC apoptosis assay, and the overexpression of caspase-3. Moreover, STX disrupted cell cycle at pre-G1 and G0/G1 phases in lung cancer. Intriguingly, STX exhibited its antitumor activity through reducing the EGFR expression. The molecular docking study revealed the potential binding interactions and affinities within the active sites of both wild-type and mutant EGFR.

**Conclusion:**

The bioprocess for optimized production, combined with the biological profiling and low cytotoxicity, substantiates the potential application of STX pigment in combating lung cancer.

## Background

Recently, microbial pigments have attracted growing scientific interest due to their diverse applications and prospective therapeutic effects. Natural pigments are more renewable and more safe than manufactured pigments, attracting industrial attention for human use [1]. These natural colorants are being used in industries including food, clothing, and cosmetics. Based on their antibacterial, antioxidant, and anticancer qualities, they have also demonstrated potential in medical applications [2]. Carotenoids, among the numerous pigments produced, have attracted particular attention because of their potential health benefits, including possible cancer-preventive characteristics and protection against oxidative stress [3]. Notably, naturally occurring carotenoids, such as phytoene, lutein, a-carotene and b-carotene, lycopene, b-cryptoxanthin, crocetin, capsanthin, fucoxanthin, neoxanthin, astaxanthin, and zeaxanthin were reported to have strong anticancer properties [4].

Staphyloxanthin (STX) belongs to a specific class of apocarotenoid triterpenoid pigments synthesized by *Staphylococcus aureus* [5]. The production of apocarotenoids results from the oxidative cleavage of C40 isoprenoids in microbes and plants. The synthesis of apocarotenoids proceeds both enzymatically, with significant reliance on carotenoid cleavage dioxygenases (CCDs), and non-enzymatically, relying on other mechanisms. The escalating detrimental effects associated with synthetic colorants have heightened the interest in apocarotenoids as a natural food colorants [6]. Despite their widespread distribution in nature, carotenoids and apocarotenoids have exceedingly low cellular concentration [7]. Currently, microbial biotechnology has enhanced the efficiency and cost-effectiveness of microbial production of carotenoids and apocarotenoids through the application of well-established fermentation strategies with specific microbial strains [8]. Industrially, carotenoids and apocarotenoids are esteemed for their significant utility attributed to their anticancer activities and health-promoting effects [5, 7, 8].

The refinement of cultivation parameters has demonstrated a capacity to enhance microbial yields, thereby augmenting the potential for bioprocessing on an industrial scale [9]. Response surface methodology (RSM) has proven to be a successful method for optimizing microbial pigment production, providing a structured framework for comprehending the intricate interactions among various variables. RSM can provide researchers with efficient exploration and modeling of the interactions among various factors and pigment yield, while markedly diminishing the resources and time necessary for optimization trials, thereby expediting the advancement of scalable production and cost-effective processes [10, 11].

Globally, lung carcinoma is a primary cause of death, and represents the second most prevalent type of cancer. Non-small cell lung cancer (NSCLC), encompassing squamous cell carcinoma and adenocarcinoma, represents the most prevalent histological subtype of lung cancer, constituting around 85% of all cases [12]. The emergence of resistance to numerous highly effective anticancer agents presents a challenge in the management of cancer. A contemporary approach for tackling cancer proliferation involves the inhibition of a trans-membrane glycoprotein, identified as epidermal growth factor receptor (EGFR), which contributes a crucial role in intracellular signaling, morphogenesis, and differentiation [13, 14]. Hence, at that time, natural products have been thoroughly examined and scrutinized for the advancement of effective anticancer agents that exhibit significant antioxidant properties while maintaining minimal toxicity to host cells [15]. In the context of the antioxidant properties of the STX pigment in countering reactive oxygen species [5], this study focuses on the anticancer assessment of the STX pigment produced by *S. aureus*.

However, the inquiry into the biological characteristics of STX pigment remains in its nascent stages and requires further exploration. Subsequently, this research seeks to systematically improve STX production through a statistical media optimization approach, employing response surface models and utilizing the Box–Wilson experimental design for both screening and validating the interactive effects of vital elements and growth factors. The evaluation of cytotoxicity to normal Vero cells was conducted to assess the safety profile. The antiproliferative activity was also determined against NSCLC cells. In addition, the cellular mechanistic analysis of anticancer activity, such as cell cycle arrest, induction of apoptosis, and EGFR inhibition, was conducted. Finally, molecular modeling was implemented to investigate putative EGFR binding modalities.

## Materials and methods

### Bacterial strains and culture media

A total of 59 *S. aureus* isolates were recovered from different clinical specimens that were collected by the staff members of Tanta University Hospitals. Mannitol salt agar (MSA; HiMedia Laboratories Pvt. Limited, Mumbai, India) was used for *S. aureus* isolation. The isolated colonies were then retrieved and presumptively identified as *S. aureus* using Gram staining examination and a panel of standard biochemical tests. The identity was verified using matrix-assisted laser desorption/ionization time-of-flight mass spectrometry (MALDI-TOF MS) [16, 17]. Nutrient agar (NA; HiMedia Laboratories Pvt. Limited, Mumbai, India) was employed to screen for the golden-yellow pigment production ability of the recovered isolates.

The optimal basal medium for the production of STX pigment has been determined through the selection of seven different experimental media (HiMedia Laboratories Pvt. Limited, Mumbai, India), including: nutrient broth, Luria-Bertani broth, tryptic soya broth, brain heart infusion broth, RPMI, glucose phosphate broth, and peptone water medium.

### Estimation of staphyloxanthin production

all the isolated *S. aureus* strains were evaluated for pigment production as previously described [18], with slight modifications. For qualitative assessment of STX, the isolates were cultivated in TSB and incubated overnight at 37 ^o^C. After that, the suspensions were adjusted to 0.5 McFarland, or roughly 10^8^ CFU/mL, and streaked on the surface of NA plates. After 24 h of incubation at 37 °C, the plates were examined for pigment production. For quantitative estimation of STX biosynthesis, all the pigmented isolates (*n* = 59) were assessed by inoculating overnight cultures in 20 mL BHI broth in 50 mL conical falcon tubes, and then incubation at 37 °C with shaking (200 rpm). Subsequently, the cell pellets of each culture were harvested after 24 h through centrifugation at 8000x g for 10 min. The collected cell pellets were then washed twice with phosphate-buffered saline (PBS) and resuspended by vortexing and pipetting in 10 mL of absolute methanol until all clumps were dispersed. To perform a complete extraction, the reaction mixture was incubated overnight in a dark environment and then centrifuged to collect the supernatants containing the extracted pigment. By measuring the absorbance of the supernatants spectrophotometrically at 456 nm, the amount of STX produced in each strain was quantified [19]. The *S. aureus* isolate with the highest STX production was selected and stored for further experimental studies.

### Molecular identification of the highest pigment-producing isolate

The genomic DNA was extracted and purified using the GeneJet Genomic DNA Purification Mini Kit (Thermo Scientific) in compliance with the manufacturer’s guidelines. A CreaCon heat cycler was used for amplification. To assess the final amplified product length, the DNA molecular weight ladder 1 kbp DNA marker (PeqGold 1Kb, Peqlab, GMH) was utilized. Amplification of the 16 S rRNA gene sequence (1500 bps) was then accomplished using 5′-AGAGTTTGATCTGGCTCAG-3′ and 5′-GGTTACCTTGTTACGACTT-3′ as forward and reverse 16 S rRNA universal primers, respectively [20]. The samples were loaded into polymerase chain reaction (PCR) equipment with 1 µL of template DNA and 1 µL of each primer added. As previously indicated, the thermal cycle was set up with the following steps: 94 °C for 6 min for the first denaturation step, 56 °C for 30 s for the annealing step, 72 °C for 2 min for the extension step, and 72 °C for 5 min for the final extension step [21]. After the PCR product was purified, the Sanger sequencing method (ABI 3730 xL DNA sequencer, Germany) was used to sequence the resulting product. The obtained sequences have been aligned and subjected to BLASTn analysis to detect sequence similarity in the National Center for Biotechnology Information (NCBI) Gene Bank database.

### Optimization of culture conditions

Prior to optimization trials, the primary production parameters that potentially regulate the bacterial growth and production of its metabolites, including factors affecting metabolic activity such as the type and concentration of carbon and nitrogen source and minerals as a micronutrient, as well as stress factors affecting bacterial growth such as pH, temperature, and incubation time, have been examined using the one-factor-at-a-time method (OFAT), as previously described [22].

A 250 mL Erlenmeyer flask holding 50 mL of test medium was used to inoculate each experimental unit (EU) with 1 mL of overnight culture. First, the effect of different fermentation media on pigment production was assessed using seven distinct experimental broths in order to determine the optimal basal medium for conducting the subsequent OFAT protocol. In the second step, the impact of the production parameters was examined to determine the optimal value of each factor. The bacterial suspension was grown in the optimal medium and incubated at different temperatures (20, 28, 37, and 55 °C), pH (5, 7, 9, and 11), and incubation times (24, 48, 72, and 96 h) under shaking (200 rpm). In order to determine the optimal nitrogen and carbon sources for STX biosynthesis, various sources of nitrogen (peptone, tryptone, beef extract, yeast extract, and (NH4)_2_SO4) and carbon (sucrose, glucose, xylose, lactose, and mannitol) were incorporated separately in the optimal fermentation medium at a concentration of 5 g/L (0.5%). For the purpose of assessing the effect of heavy metals on pigment production, different metal ions (CaCl_2_, ZnCl_2_, MnCl_2_, FeSO_4_, MgSO_4_, and CuSO_4_) were tested at a concentration of 10 mM. For each factor assessment, the flasks were incubated at the optimal value of other factors under shaking (200 rpm), and the effect of these factors was determined by measuring the absorbance of the pigment extracted in each EU at 456 nm.

### Multifactorial optimization of pigment production using response surface methodology (RSM)

For assessing the optimal combination and interactive consequences of crucial process variables on the higher yield of STX, a response surface method with central composite design (CCD) was utilized [23–25]. The experimental design and analysis were conducted using Design-Expert^®^ 6.0.8 statistical software (Stat-Ease, Minneapolis, MN, USA). In order to establish the experimental design, RSM was employed utilizing six variables, where each independent variable was evaluated at three different levels (+ 1, 0, -1) for the first high level, the central level, and the first low level, respectively, as presented in Table 1. Consequently, an overall of CCD 86 experimental designs were set for examination of the response of the pigment production. Each experimental unit was run in triplicate, and the average amounts of the produced pigment were considered the responses (Y) or the dependent variables. The second-degree polynomial equation, which included all terms, was used to compute the predicted response as follows:

**Table 1:** Experimental factors and level of minimum and maximum range for statistical screening using central composite design (CCD)

Factors	Variables	Unit	Production level		
			Minimum (-1)	Intermediate (0)	Maximum (+ 1)
X1	Temperature	-	30	37	44
X2	pH	-	5	7	9
X3	Mannitol	g %	0.5	1.5	2.5
X4	Peptone	g %	0.5	1.5	2.5
X5	Incubation time	hr	24	48	72
X6	ZnCl2	mM	10	20	30

$$\:Y=\beta\:0\:+\:\sum\:\beta\:iXi\:+\:\sum\:\:\beta\:iiX2\:i\:+\sum\:\:\beta\:ijXiXj$$

Where the intercept coefficient is represented by 𝛽0, the linear effect coefficient is represented by 𝛽𝑖, the quadratic impact coefficient by 𝛽𝑖𝑖, the interaction coefficient effect is represented by 𝛽𝑖𝑗, and the 𝑖th linear coefficient is 𝛽𝑖. Input variables 𝑋𝑖𝑋𝑗 impact the response variable 𝑌.

The analysis of variance was used to conduct the model’s statistical and numerical analysis (ANOVA). Fisher’s 𝐹-test, its corresponding probability 𝑃(𝐹), correlation coefficient 𝑅, and determination coefficient 𝑅^2^ were used to assess the statistical significance of the model, which explains the polynomial model’s quality. Response surface curves were made for each variable and three-dimensional contour plots were used to depict the quadratic models. The model was validated at a particular level of improved critical process variables for increased pigment synthesis.

### Extraction and purification of staphyloxanthin pigment

The best STX pigment-producing strain, *S. aureus* A2, was grown in a 250-mL Erlenmeyer flask containing BHI broth with the optimal growth parameters, and incubated at 37 °C for 48 h with shaking (200 rpm). After incubation, the cell pellets were collected through centrifugation at 8000 x g for 10 min and mixed with methanol in a ratio of 4:1 (solvents/pellets, v/w). The orange-colored supernatant was centrifuged at 10,000 x g for 15 min at 4 °C, and the pellets were exhausted with solvent multiple times until the pellets lost color [19]. The collected supernatant was purified using silica gel column chromatography (60–120 mesh size), as previously described [26]. First, the sample was defatted by eluting with n-hexane, followed by elution with absolute chloroform to dispel the impurities. Second, the polarity of the mobile phase was increased by the addition of ethanol, starting from a fraction of chloroform: ethanol combination at a ratio of 99:1 (v/v). The golden-yellow-colored eluted fractions were collected from the column. Finally, the purified pigment was evaporated in a porcelain dish at 40 °C, and the pigment powder was stored in darkness for further investigation [27].

### Characterization of staphyloxanthin pigment

#### Thin-layer chromatography (TLC)

To select the suitable solvent, a variety of solvent systems were examined. The TLC plate was spotted with the purified pigment which was completely dissolved in methanol. The plate was then placed within a container fully saturated with a methylene chloride: ethanol combination at a ratio of 9.5: 0.5 (v/v). The retardation factor (Rf) was measured after the plate was removed from the container whenever the solvent migration had reached its top limit [28]. Ultraviolet (UV) analysis with a UV–vis spectrophotometer was employed to confirm the presence of the purified pigment.

#### Fourier-Transform infrared spectroscopy (FTIR)

The FTIR spectrum was used to determine the functional groups present in the purified pigment. In brief, 100 mg of KBr and 1 mg of the dried pigment were mixed to make a homogeneous pellet, which was subsequently submitted for analysis using an FTIR spectrometer (FTIR SPECTROMETER 4100 JASCO-JAPAN) with a resolution of 4 cm-1 from 4000 to 400 cm-1 [29].

### Mass spectroscopy

Thin layer chromatography-mass spectroscopy (TLC-MS) was conducted using Advion compact mass spectrometer (CMS) NY-USA. The flow rate ranged from 10 µL/min to 1 mL/min. The result was expressed as a positive mode (M + H^+^) [5].

### Safety profile analysis of the purified staphyloxanthin pigment

The MTT assay was utilized to test the produced pigment’s cytotoxicity against Vero cells (ATCC, USA) [30]. In brief, the cells were cultured in Dulbecco’s Modified Eagle Medium (DMEM) supplemented with 100 µg/mL streptomycin, 100 U/mL penicillin, and 10% fetal bovine serum (FBS). Vero cells were seeded in a 96-well microplate at a density of 5 × 10^3^ cells/well in 100 µl of complete growth media. They were then cultured overnight at 37 °C with 5% CO_2_ to create a semi-confluent layer. Then, cells were treated in triplicate at 37 °C in a CO_2_ atmosphere with varying concentration of the pigment (0, 31, 62.5, 125, 250, 500, and 1000 µg/mL). After the incubation period, the medium was removed and the cells were washed with PBS for cleaning. After dissolving 100 µl of MTT (0.5 mg/mL) in serum-containing DMEM medium, each well was incubated for 4 h. In order to dissolve the formazan crystals, 100 µl of DMSO was added to each well after mixing. The optical density (OD) of every well was measured at 492 nm and a reference wavelength of 630 nm using a microplate reader (Model 4300; Chromate Instrument, Awareness technologies, Inc., Palm City, USA). The percentage of cell viability was calculated by dividing the OD test by the OD control and then multiplying the result by 100.

### Biological evaluation

#### In vitro anticancer activity of staphyloxanthin

The MTT assay was used to evaluate the cytotoxicity of STX against A549 cells lung cancer, as previously described [31]. The cells are cultivated at 37 °C in a humidified incubator with 5% CO_2_ in RPMI 1640 media supplemented with 10% FBS (Gibco, USA), 100 U/mL penicillin, and 100 µg/mL streptomycin sulfate (Lonza, Belgium). To create a semi-confluent layer, 100 µl of cells with plating densities of 5 × 10^3^ cells per well were plated into 96-well microtiter plates. The cells were then cultured overnight at 37 °C with 5% CO_2_. After adding the pigment with varying concentrations (0, 31, 62.5, 125, 250, 500, and 1000 µg/mL), the plates were incubated at 37 °C, 95% air, and 5% CO_2_ for 48 h. Following that, 100 µL of MTT (0.5 mg/mL) is added to each well, and the plates were incubated for 4 h. In order to dissolve the formazan crystals, 100 µl of DMSO was added to each well after mixing. An automatic reader (Model 4300; Chromate Instrument, Awareness technology, Inc., Palm City, USA) was used to measure the absorbance at 492 nm and a reference wavelength at 630 nm. Using the seven absorbance measurements, the percentage growth of the treated cells relative to the untreated control cells was calculated.

### Detection of necrosis and apoptosis using confocal laser scanning microscopy (CLSM)

Using the double-staining approach with acridine orange (AO) and propidium iodide (PI), the impact of STX on the viability of A549 lung cancer cells was evaluated [32]. In essence, A549 cells were seeded at a density of 1 × 10^5^ cells/well in a 12-well plate, incubated for overnight, and then treated with IC50 concentration of STX for 24 h. Following medium removal and PBS washing, the cells were then treated with AO/PI stains and allowed to sit at room temperature for 15 min in a dark environment. Stained A549 cells were washed and viewed at 10× and 20× magnification using a fluorescence microscope (DMi8; Leica Microsystems).

### Annexin V-FITC apoptosis assay

Annexin V-FITC apoptosis detection kit (Abcam Inc., Cambridge Science Park, Cambridge, UK), combined with two fluorescent avenues flow cytometry, was applied to determine apoptosis and necrosis cell populations. In a humidified, 5% (v/v) CO_2_ environment, cells were kept in DMEM media supplemented with 100 U/mL penicillin, 100 µg/mL streptomycin, and 10% heat-inactivated fetal bovine serum at 37 °C. Following a 24-h STX treatment, 10^5^ cells were trypsinized and then rinsed twice with ice-cold PBS (pH 7.4). Subsequently, in accordance with the manufacturer’s instructions, cells were incubated for 30 min at room temperature in dark with 0.5 ml of Annexin V-FITC/PI solution. The ACEA Novocyte™ flowcytometer (ACEA Biosciences Inc., San Diego, CA, USA) was utilized to inject cells. The FL1 and FL2 signal detectors were used to detect the fluorescent signals for FITC and PI, which are λex/em 488/530 nm for FITC and λex/em 535/617 nm for PI. ACEA NovoExpress™ software (ACEA Biosciences Inc., San Diego, CA, USA) was used to calculate positive FITC and/or PI cells by quadrant analysis after collection of 12,000 events per sample [33, 34].

### Cellular mechanistic analysis

Flow cytometry was employed to investigate the effect of STX on the cell cycle [33, 34]. A549 cells were treated with the purified pigment prior to being cultivated for 24 h at 37 °C in 5% CO_2_. The cell pellets (10^5^ cells) were collected after trypsinization and centrifugation. They are then washed twice with ice-cold PBS (pH 7.4). For fixation, the cells were resuspended in 2 mL of 60% ice-cold ethanol, and then incubated for 1 h at 4ºC. After two PBS (pH 7.4) washes, the fixed cells were resuspended in 1 mL of PBS containing 10 µg/mL propidium iodide (PI) and 50 µg/mL RNAase A. A FL2 (λex/em 535/617 nm) signal detector was used to evaluate the DNA content of cells following a 20-min dark incubation at 37 °C. For every sample, 12,000 events were gathered. Cell cycle distribution was calculated using ACEA NovoExpress™ software.

### Assessment of gene expression using real-time PCR

According to earlier reports, the purified pigment’s effect on the expression of EGFR and caspase-3 genes in A549 lung cancer cells was assessed [32, 35]. Total RNA was extracted pursuant to the kit’s instructions (Roche Diagnostic GmbH, Germany). The NanoDrop™ 2000c Spectrophotometer (Thermo Scientific) was used to determine the purity and concentration of extracted RNA samples. Using the QuantiTects Reverse Transcription Kit (Cat. No.: MB305-0050, Qiagen, USA) and following the manufacturer’s instructions, the extracted RNA was reverse transcribed into complementary DNA (cDNA). The Bio-Rad CFX OPUS 96 platform and SYBR Green qPCR master mix (Xpert Fast SYBR (uni), Cat. No. # GE20.100, Porto, Portugal) were used to determine gene expression levels of the caspase-3 and EGFR genes, with GAPDH serving as the housekeeping gene. The final volume was 20 µl and included 2 µl of cDNA, 2 µl of forward and reverse primers (0.3–0.5 µM), 10 µl of master mix, and 6 µl of nuclease-free water. The primers used in real-time PCR are listed in Table 2.

**Table 2:** Primer sequence involved in real-time PCR

Primer name	Primer sequence
Caspase-3 gene expression	
Caspase-3	F: 5’-TGTTTGTGTGCTTCTGAGCC-3’ R: 5’-CACGCCATGTCATCATCAAC-3’
Glycerol 3 Phosphate Dehydrogenase (GPDH) Human qPCR Primer Pair	F: 5’-ATACCTGCCAGGGCACAAGTTG-3’ R: 5’-CAGTGGCGTTTGCCTTCAGATG-3’
EGFR gene expression	
GAPDH	F: 5’-TGCACCACCAACTGCTTAGC-3’ R: 5’-GCAGGGATGATGGTTCTGGAG-3’
EGFR	F: 5’-TGCGTCTCTTGCCGGAAT-3’ R: 5’-GGCTCACCCTCCAGAAGGTT-3’

### Docking protocol

The interactions of STX pigment with EGFR^WT^, EGFR^T790M^, and EGFR^TL858R^ to analyze their inhibitory activities was investigated. The docking process involved placing the pigment within the active sites of the target enzymes. The binding modes and orientations were analyzed using EGFR^WT^ (PDB code: 1M17) [36], EGFR^T790M^ (PDB code: 2JIV) [37], and EGFR^L858R^ (PDB code: 4LQM) structures [38]. The MOE-Dock software version 2020.09 was utilized for analysis. The 2D configuration of the STX pigment was depicted using Chem. Draw. All residues were removed and polar hydrogens were added to the protein chains under study using the protonate 3D protocol in MOE with its default settings. This produced valuable protonation states for the simulations of molecular docking. Protein Data Bank access was granted for the co-crystallized structures of EGFR^WT^, EGFR^T790M^, and EGFR^TL858R^ with their ligands, with PDB codes of 1M17, 2JIV, and 2JIV, respectively. The partial charges were computed automatically after all minimizations were carried out using MOE with MMFF94x force field until a Root Mean Square Deviation (RMSD) gradient of 0.05 kcal mol^− 1^ Å^−1^. Convolutional analysis of ligands was produced from a single 3D conformation by adding hydrogens and partial charges and carrying out a methodical search that produced each potential combination of dihedral angles for each ligand [39, 40]. The London-dG scoring function and Triangle Matcher placement method were employed to generate the docking pose.Each ligand was given consideration for a maximum of five refinements out of thirty conformers (poses). The most desirable (least) free binding energy & distance, RMSD (≤ 2), and interactions with the amino acids in the binding cavity target site were the criteria used to select the optimal ligand conformation [13, 41].

### Statistical analysis

The degree of variability of the results was determined as mean ± standard deviation. The F-test and statistical significance were conducted using a one-way ANOVA test. The multifactorial optimization results were analyzed using Design-Expert^®^ software (version 6.0.8).

## Results

### Isolation and identification of staphyloxanthinproducing bacteria

The current investigation yielded a total of 59 *S. aureus* isolates from various clinical specimens obtained from Tanta Hospitals. The bacterial strains were tentatively recognized as *S. aureus* based on their biochemical and morphological features. Gram staining demonstrated a distinct grape-like cluster shape, indicating the morphology of *S. aureus*. The *S. aureus* isolates exhibited yellow colonies on the mannitol salt agar. Additional biochemical analysis at the species level confirmed that all isolates exhibited catalase, coagulase, and DNase activity, and also demonstrated beta-hemolysis on blood agar. The production of STX was validated through detection of golden-yellow color on NA plates.

### Estimation of staphyloxanthin production

**Fig. 1:**
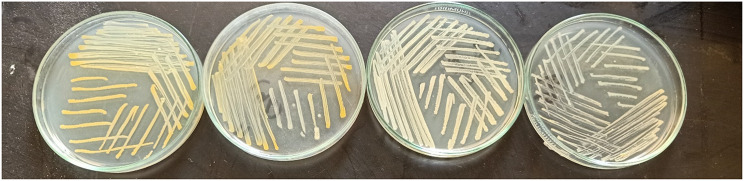
Variable STX pigmentation of the screened *S. aureus* isolates

The amount of STX produced by each strain varied, however all *S. aureus* strains were quantitatively examined for the production of STX pigment. The colonies’ distinct golden-yellow coloring, as seen in Fig. 1, was demonstrated during the screening of *S. aureus* isolates, suggesting a high potential for generating STX. Additionally, spectrophotometrically assessing the absorbance of the methanolic extracts of the tested isolates at 456 nm allowed for the quantification of STX synthesis. The isolates were categorized into three groups according to their optical densities (OD): intensely pigmented (OD_456_ > 0.2), moderately pigmented (OD_456_ = 0.1–0.2), and weakly pigmented (OD_456_ < 0.1). It was observed that 3 (5.08%) of the recovered isolates were extremely pigmented, 49 (81.05%) were moderately pigmented, and 7 (11.86%) were weakly pigmented. The results revealed that the isolate A2 showed the highest STX production with optical density of 0.215. Therefore, it was selected for further study.

### Gene alignment in Genbank (Blast)

**Fig. 2:**
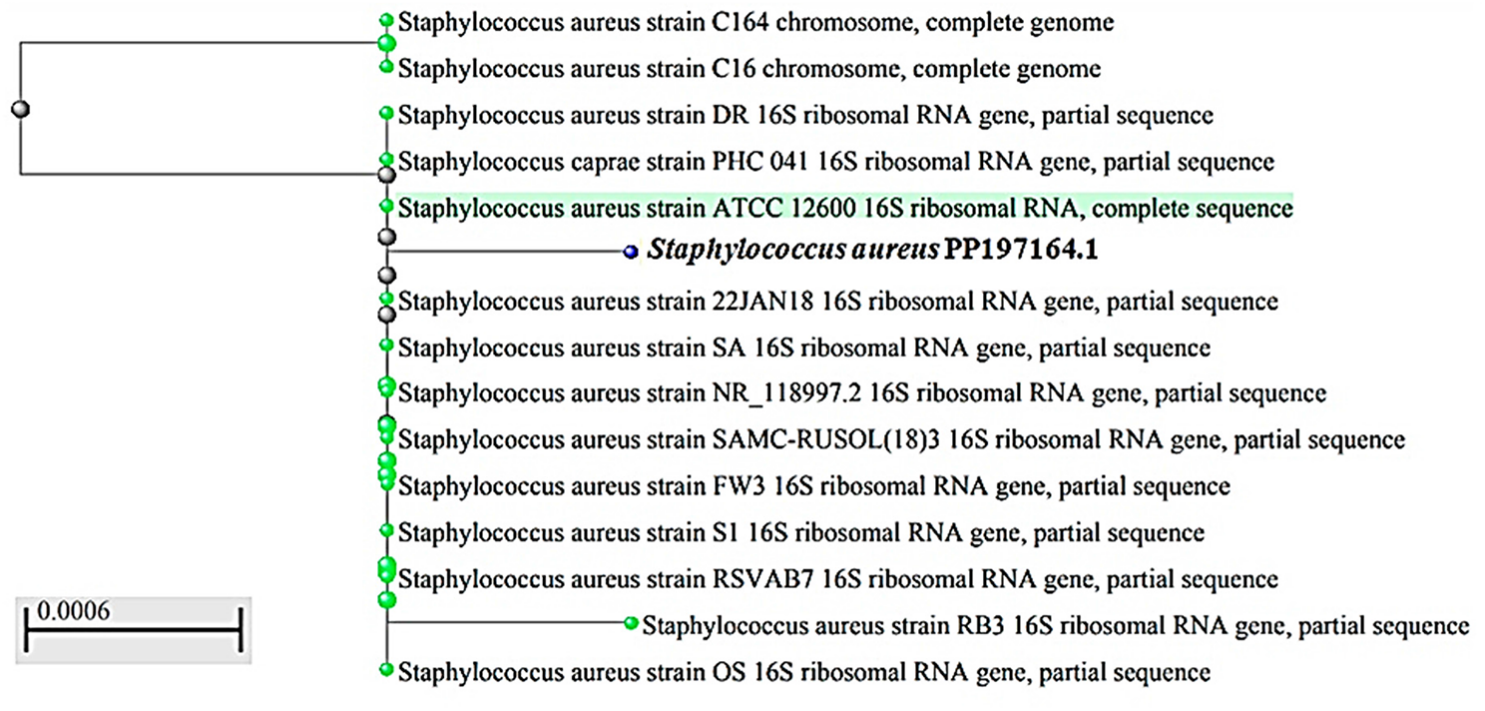
Phylogenetic analysis of *S. aureus* A2 isolate based on BLAST homology of the 16 S rRNA sequence

16 S rRNA sequencing was used to further corroborate the molecular identity of the *S. aureus* A2 strain that produced the most STX. The acquired nucleotide sequence was submitted into the Gene Bank database and assigned the accession number (PP197164) to *S. aureus* A2. (https://www.ncbi.nlm.nih.gov/nuccore/PP197164.1/). The Blast program was used to analyze the nucleotide sequence. The isolate A2 was identified by the neighbor-joining phylogeny analysis as belonging to the same group and being closely related to *S. aureus* based on the phylogenetic tree, as illustrated in Fig. 2. There was a 99.93% similarity between the nucleotide alignment in GenBank (Blast) and *S. aureus* strain OS with accession No. MN508958.1.

### Optimization of culture conditions (OFAT protocol)

#### Determination of the optimal basal medium for the pigment production

**Fig. 3:**
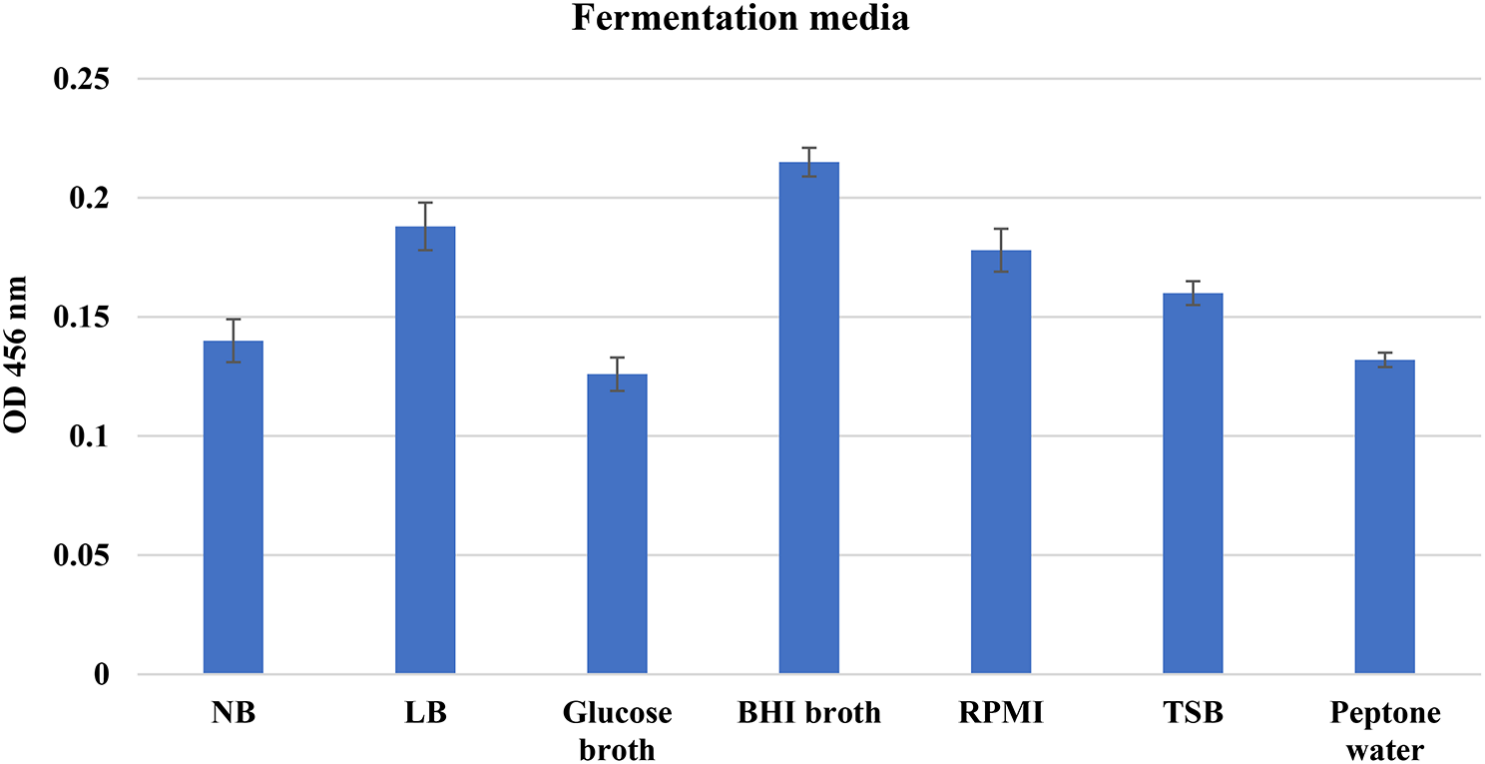
The impact of various fermentation media on STX yield from the selected *S. aureus* S53 isolate. Data represented as mean ± SD (*n* = 3). NB; nutrient broth, LB; Luria-Bertani broth, TSB; tryptic soya broth, BHI; brain heart infusion broth

Seven distinct experimental broths were used to evaluate the effect of various fermentation media on pigment production by *S. aureus* A2 isolate in order to identify the optimal basal medium. As shown in Fig. 3, the results showed that the selected organism thrived most successfully in BHI medium (OD456 = 0.215), which served as a control basal medium. Notably, the highest production was achieved by employing the latter medium, which was 1.7 times greater than that of the glucose broth medium and 1.6 times more than that of the peptone water medium.

### Screening of the optimal growth parameters for pigment production

**Fig. 4:**
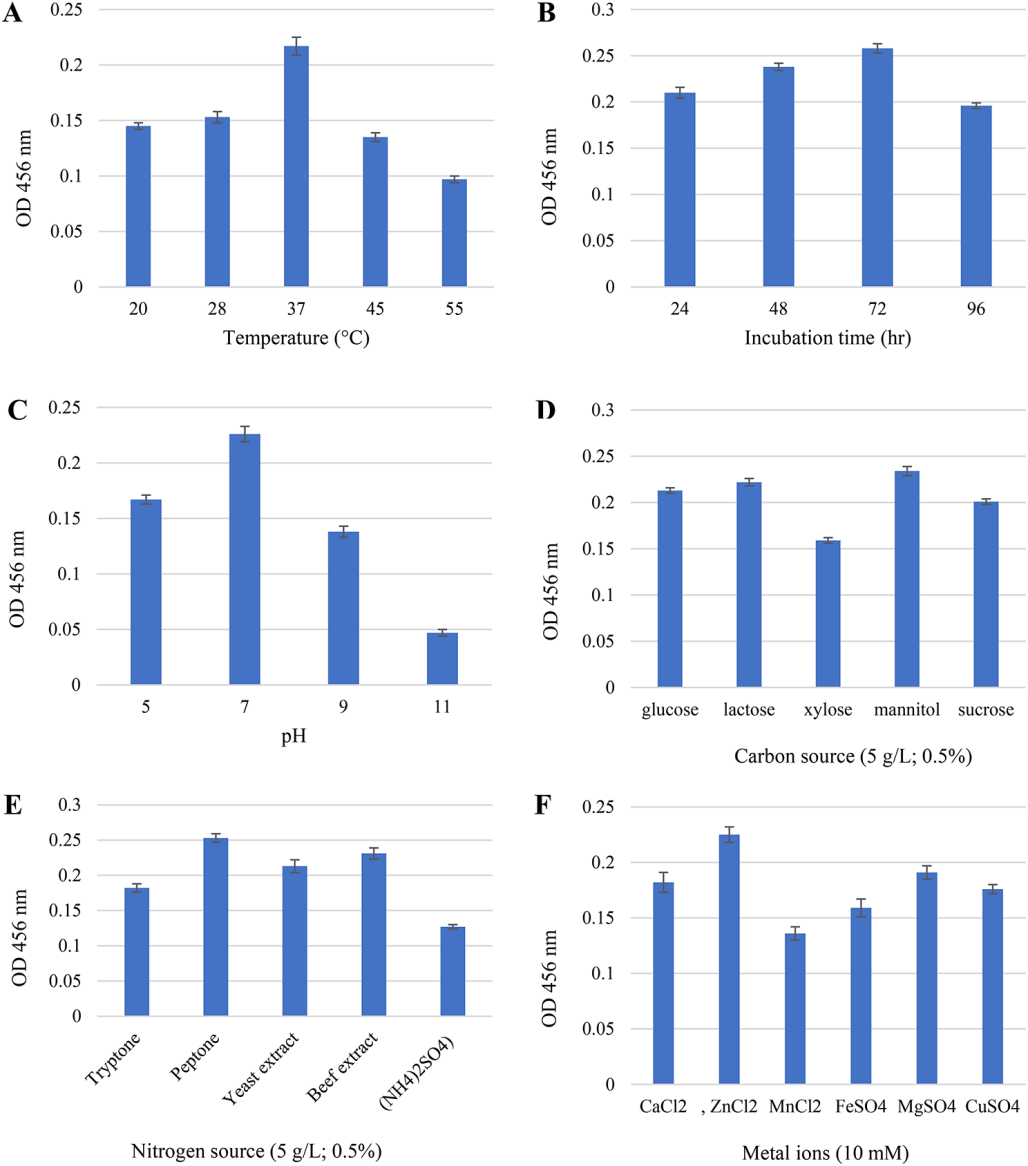
The impact of different growth parameters on STX production; (**A**) temperature, (**B**) incubation time, (**C**) pH, (**D**) carbon source, (**E**) nitrogen source, and (**F**) metal ions. Data represented as mean ± SD (*n* = 3)

Each growth factor under investigation demonstrated substantial variation in STX production, as shown in Fig. 4. Different temperatures ranging from 20 °C to 55 °C were used to assess the incubation temperature on STX yield. As illustrated in Fig. 4(A), the pigment production maximized at 37 °C (OD_456_ = 0.217) and showed lower yields at higher and lower temperatures. The selected *S. aureus* A2 isolate was cultivated in BHI broth at 37 °C to investigate the effect of the incubation period. According to our results, the highest STX production (OD_456_ = 0.258) was observed after 72 h, while extending the incubation period to 96 h significantly decreased the pigment yield (OD_456_ = 0.196) (*P* < 0.05), as illustrated in Fig. 4(B). Considering the production medium’s pH is essential to microbial growth, the effect of the pH on pigment production was assessed. The optimal incubation duration was set at 72 h, and the STX yield was determined at pH values ranging from 5 to 11 using HCL (1 N) and NaOH (1 N). STX production was determined to be maximum at pH 7 (OD_456_ = 0.226) and lowest at pH 11 (OD_456_ = 0.047), as shown in Fig. 4(C). Additionally, the effect of carbon sources on the production of STX was determined. Mannitol (OD456 = 0.234) was the most effective carbon source, as illustrated in Fig. 4(D). Lactose (OD_456_ = 0.222) and glucose (OD_456_ = 0.213) were close behind. However, xylose was found to be an inappropriate carbon source for STX synthesis considering that it reduced STX production by 1.5 times (OD_456_ = 0.159), compared to mannitol. Since both organic and inorganic nitrogen sources are essential for the formation of pigment, the impact of various nitrogen sources on STX production was also assessed. Upon utilizing peptone as a nitrogen source, STX yielded the greatest yield (OD_456_ = 0.235), followed by yeast extract (OD_456_ = 0.213) and beef extract (OD_456_ = 0.231), while, tryptone and (NH4)2SO_4_ showed a decrease in STX production by 1.3 and 1.8 times (OD_456_ = 0.182 and 0.127), respectively, compared to peptone (Fig. 4(E)). The impact of several metal ions on pigment formation was evaluated at a final concentration of 10 mM. As illustrated in Fig. 4(F), the highest production of STX was achieved using ZnCl_2_ (OD_456_ = 0.225), followed by MgSO_4_ (OD_456_ = 0.191). The lowest production was achieved when MnCl_2_ (OD_456_ = 0.136) was used.

### Optimization of staphyloxanthin production using response surface methodology

An essential aspect of enhancing and optimizing STX production involves the mathematical and statistical examination of multivariate data acquired using response surface methodology. The most well-established approach for biological molecules production, particularly in submerged fermentation, is the response surface method, a software-based statistical design. This study employed a central composite model to produce STX by conducting an 86-run experimental design on the *S. aureus* A2 strain, with the highest STX production. The matrix comprised six components, each with three levels (-1, 0, and + 1), and three replicates at the center point. Table 3 displays the independent factors using a coded matrix, along with the corresponding responses, as well as the calculated and anticipated values for STX production. Variations in experimental circumstances over the course of the 86 runs led to fluctuations in pigment yield, underscoring the necessity for statistical optimization of fermentation parameters as an alternative to the conventional approaches. The most optimal STX production was observed in run number 50, with a yield of OD_456_ = 0.328, which is approximately 1.5-fold greater than the yield obtained with un-optimized media (BHI: OD_456_ = 0.215). The most effective conditions for achieving the highest production of STX were a pH of 5, a temperature of 37 °C, an incubation time of 48 h, a mannitol concentration of 2.5%, a peptone concentration of 1.5%, and a ZnCl2 concentration of 20 mM. The squared regression coefficient (R^2^) determination demonstrated a strong level of accuracy for the model, with a precise value of 0.8748. The observed association was statistically significant, providing strong evidence for the accuracy of the current model in predicting STX yield. The second-order equation representing the final outcome, taking into account the actual factors, is as follows:

**Table 3:** Design of optimization trials of the CCD for independent variables and responses by the highly staphyloxanthin-producing *S. aureus* A2 isolate

Run	A. Temp	B. pH	C. Mannitol	D. Peptone	E. Incu. time	F. ZnCl_2_	O.D_456_ nm Actual value	O.D_456_ nm Predicted value	Residual
1	44 (+ 1)	9(+ 1)	0.5(-1)	0.5(-1)	72(+ 1)	30(+ 1)	0.238	0.2106	0.0274
2	37(0)	7(0)	1.5(0)	1.5(0)	72(+ 1)	20(0)	0.207	0.2164	-0.0094
3	37(0)	7(0)	1.5(0)	1.5(0)	48(0)	20(0)	0.246	0.2488	-0.0028
4	44(+ 1)	9(+ 1)	0.5(-1)	2.5(+ 1)	72(+ 1)	30(+ 1)	0.219	0.2465	-0.0275
5	44(+ 1)	9(+ 1)	2.5(+ 1)	2.5(+ 1)	72(+ 1)	10(-1)	0.244	0.2331	0.0109
6	44(+ 1)	5(-1)	0.5(-1)	0.5(-1)	72(+ 1)	10(-1)	0.166	0.1610	0.005
7	30(-1)	5(-1)	2.5(+ 1)	2.5(+ 1)	72(+ 1)	30(+ 1)	0.231	0.2424	-0.0114
8	30(-1)	9(+ 1)	0.5(-1)	0.5(-1)	24(-1)	10(-1)	0.159	0.1651	-0.0061
9	30(-1)	9(+ 1)	2.5(+ 1)	0.5(-1)	72(+ 1)	30(+ 1)	0.298	0.2848	0.0132
10	30(-1)	9(+ 1)	2.5(+ 1)	2.5(+ 1)	24(-1)	10(-1)	0.238	0.2577	-0.0197
11	37(0)	7(0)	1.5(0)	1.5(0)	48(0)	20(0)	0.246	0.2488	-0.0028
12	30(-1)	5(-1)	2.5(+ 1)	2.5(+ 1)	24(-1)	10(-1)	0.232	0.2399	-0.0079
13	44(+ 1)	9(+ 1)	0.5(-1)	2.5(+ 1)	72(+ 1)	10(-1)	0.208	0.2283	-0.0203
14	44(+ 1)	5(-1)	2.5(+ 1)	2.5(+ 1)	24(-1)	30(+ 1)	0.238	0.2190	0.019
15	37(0)	7(0)	1.5(0)	1.5(0)	48(0)	20(0)	0.246	0.2488	-0.0028
16	30(-1)	5(-1)	2.5(+ 1)	0.5(-1)	24(-1)	10(-1)	0.169	0.1689	0.0001
17	44(+ 1)	9(+ 1)	0.5(-1)	0.5(-1)	24(-1)	30(+ 1)	0.174	0.1586	0.0154
18	44(+ 1)	9(+ 1)	2.5(+ 1)	0.5(-1)	24(-1)	10(-1)	0.176	0.1671	0.0089
19	30(-1)	9(+ 1)	2.5(+ 1)	0.5(-1)	24(-1)	30(+ 1)	0.178	0.2066	-0.0286
20	37(0)	7(0)	1.5(0)	1.5(0)	48(0)	20(0)	0.246	0.2488	-0.0028
21	44(+ 1)	7(0)	1.5(0)	1.5(0)	48(0)	20(0)	0.261	0.2617	-0.0007
22	44(+ 1)	9(+ 1)	2.5(+ 1)	0.5(-1)	72(+ 1)	10(-1)	0.212	0.2070	0.005
23	30(-1)	5(-1)	0.5(-1)	0.5(-1)	72(+ 1)	10(-1)	0.153	0.1452	0.0078
24	44(+ 1)	5(-1)	0.5(-1)	0.5(-1)	72(+ 1)	30(+ 1)	0.179	0.1857	-0.0067
25	37(0)	7(0)	1.5(0)	1.5(0)	48(0)	20(0)	0.246	0.2488	-0.0028
26	30(-1)	9(+ 1)	2.5(+ 1)	2.5(+ 1)	72(+ 1)	30(+ 1)	0.309	0.2885	0.0205
27	44(+ 1)	5(-1)	2.5(+ 1)	0.5(-1)	72(+ 1)	10(-1)	0.188	0.1813	0.0067
28	30(-1)	5(-1)	2.5(+ 1)	2.5(+ 1)	72(+ 1)	10(-1)	0.212	0.2188	-0.0068
29	44(+ 1)	9(+ 1)	0.5(-1)	0.5(-1)	72(+ 1)	10(-1)	0.185	0.1815	0.0035
30	30(-1)	5(-1)	0.5(-1)	2.5(+ 1)	24(-1)	10(-1)	0.218	0.2324	-0.0144
31	37(0)	7(0)	1.5(0)	1.5(0)	48(0)	20(0)	0.246	0.2488	-0.0028
32	44(+ 1)	5(-1)	2.5(+ 1)	2.5(+ 1)	72(+ 1)	30(+ 1)	0.218	0.2213	-0.0033
33	30(-1)	7(0)	1.5(0)	1.5(0)	48(0)	20(0)	0.281	0.2746	0.0064
34	44(+ 1)	9(+ 1)	2.5(+ 1)	0.5(-1)	72(+ 1)	30(+ 1)	0.214	0.2242	-0.0102
35	37(0)	7(0)	1.5(0)	1.5(0)	48(0)	20(0)	0.246	0.2488	-0.0028
36	44(+ 1)	9(+ 1)	0.5(-1)	2.5(+ 1)	24(-1)	10(-1)	0.254	0.2517	0.0023
37	44(+ 1)	5(-1)	2.5(+ 1)	0.5(-1)	24(-1)	10(-1)	0.151	0.1654	-0.0144
38	30(-1)	5(-1)	2.5(+ 1)	2.5(+ 1)	24(-1)	30(+ 1)	0.209	0.2326	-0.0236
39	30(-1)	9(+ 1)	2.5(+ 1)	2.5(+ 1)	24(-1)	30(+ 1)	0.282	0.2547	0.0273
40	37(0)	7(0)	0.5(-1)	1.5(0)	48(0)	20(0)	0.285	0.2984	-0.0134
41	37(0)	7(0)	1.5(0)	1.5(0)	48(0)	20(0)	0.246	0.2488	-0.0028
42	30(-1)	5(-1)	0.5(-1)	0.5(-1)	24(-1)	30(+ 1)	0.155	0.1561	-0.0011
43	44(+ 1)	5(-1)	2.5(+ 1)	2.5(+ 1)	72(+ 1)	10(-1)	0.237	0.2193	0.0177
44	44(+ 1)	9(+ 1)	2.5(+ 1)	0.5(-1)	24(-1)	30(+ 1)	0.137	0.1535	-0.0165
45	37(0)	5(-1)	1.5(0)	1.5(0)	48(0)	20(0)	0.227	0.2140	0.013
46	30(-1)	9(+ 1)	2.5(+ 1)	0.5(-1)	24(-1)	10(-1)	0.231	0.1986	0.0324
47	44(+ 1)	9(+ 1)	2.5(+ 1)	2.5(+ 1)	24(-1)	10(-1)	0.242	0.2377	0.0043
48	30(-1)	9(+ 1)	2.5(+ 1)	2.5(+ 1)	72(+ 1)	10(-1)	0.256	0.2605	-0.0045
49	44(+ 1)	9(+ 1)	2.5(+ 1)	2.5(+ 1)	72(+ 1)	30(+ 1)	0.244	0.2394	0.0046
**50**	**37(0)**	**7(0)**	**2.5(+ 1)**	**1.5(0)**	**48(0)**	**20(0)**	**0.328**	**0.3089**	**0.0191**
51	30(-1)	5(-1)	0.5(-1)	2.5(+ 1)	24(-1)	30(+ 1)	0.259	0.2370	0.022
52	37(0)	9(+ 1)	1.5(0)	1.5(0)	48(0)	20(0)	0.228	0.2353	-0.0073
53	44(+ 1)	5(-1)	0.5(-1)	2.5(+ 1)	72(+ 1)	30(+ 1)	0.248	0.2336	0.0144
54	44(+ 1)	5(-1)	0.5(-1)	2.5(+ 1)	24(-1)	10(-1)	0.274	0.2672	0.0068
55	44(+ 1)	9(+ 1)	0.5(-1)	2.5(+ 1)	24(-1)	30(+ 1)	0.258	0.2390	0.019
56	37(0)	7(0)	1.5(0)	1.5(0)	48(0)	30(+ 1)	0.217	0.1941	0.0229
57	44(+ 1)	9(+ 1)	2.5(+ 1)	2.5(+ 1)	24(-1)	30(+ 1)	0.189	0.2131	-0.0241
58	37(0)	7(0)	1.5(0)	0.5(-1)	48(0)	20(0)	0.236	0.2359	1E-04
59	44(+ 1)	5(-1)	0.5(-1)	2.5(+ 1)	72(+ 1)	10(-1)	0.218	0.2198	-0.0018
60	30(-1)	9(+ 1)	2.5(+ 1)	0.5(-1)	72(+ 1)	10(-1)	0.218	0.2460	-0.028
61	37(0)	7(0)	1.5(0)	1.5(0)	48(0)	20(0)	0.246	0.2488	-0.0028
62	30(-1)	5(-1)	2.5(+ 1)	0.5(-1)	24(-1)	30(+ 1)	0.197	0.1724	0.0246
63	30(-1)	5(-1)	0.5(-1)	2.5(+ 1)	72(+ 1)	10(-1)	0.209	0.1925	0.0165
64	37(0)	7(0)	1.5(0)	1.5(0)	48(0)	10(-1)	0.166	0.1832	-0.0172
65	44(+ 1)	5(-1)	0.5(-1)	0.5(-1)	24(-1)	10(-1)	0.151	0.1639	-0.0129
66	30(-1)	9(-1)	0.5(-1)	0.5(-1)	72(+ 1)	30(+ 1)	0.269	0.2444	0.0246
67	30(-1)	5(-1)	2.5(+ 1)	0.5(-1)	72(+ 1)	30(+ 1)	0.218	0.2267	-0.0087
68	30(-1)	5(-1)	0.5(-1)	0.5(-1)	24(-1)	10(-1)	0.146	0.1406	0.0054
69	30(-1)	5(-1)	0.5(-1)	0.5(-1)	72(+ 1)	30(+ 1)	0.183	0.1915	-0.0085
70	30(-1)	9(+ 1)	0.5(-1)	2.5(+ 1)	24(-1)	30(+ 1)	0.228	0.2538	-0.0258
71	44(+ 1)	5(-1)	0.5(-1)	2.5(+ 1)	24(-1)	30(+ 1)	0.241	0.2501	-0.0091
72	30(-1)	5(-1)	0.5(-1)	2.5(+ 1)	72(+ 1)	30(+ 1)	0.216	0.2280	-0.012
73	30(-1)	9(+ 1)	0.5(-1)	0.5(-1)	24(-1)	30(+ 1)	0.162	0.1849	-0.0229
74	44(+ 1)	5(-1)	2.5(+ 1)	2.5(+ 1)	24(-1)	10(-1)	0.234	0.2480	-0.014
75	37(0)	7(0)	1.5(0)	1.5(0)	24(-1)	20(0)	0.216	0.2009	0.0151
76	37(0)	7(0)	1.5(0)	1.5(0)	48(0)	20(0)	0.246	0.2488	-0.0028
77	44(+ 1)	5(-1)	2.5(+ 1)	0.5(-1)	24(-1)	30(+ 1)	0.164	0.1473	0.0167
78	30(-1)	9(+ 1)	0.5(-1)	2.5(+ 1)	72(+ 1)	30(+ 1)	0.266	0.2688	-0.0028
79	37(0)	7(0)	1.5(0)	2.5(+ 1)	48(0)	20(0)	0.295	0.2894	0.0056
80	30(-1)	9(+ 1)	0.5(-1)	2.5(+ 1)	72(+ 1)	10(-1)	0.238	0.2290	0.009
81	44(+ 1)	5(-1)	0.5(-1)	0.5(-1)	24(-1)	30(+ 1)	0.148	0.1577	-0.0097
82	44(+ 1)	9(+ 1)	0.5(-1)	0.5(-1)	24(-1)	10(-1)	0.163	0.1604	0.0026
83	30(-1)	9(+ 1)	0.5(-1)	0.5(-1)	72(+ 1)	10(-1)	0.178	0.1937	-0.0157
84	44(+ 1)	5(-1)	2.5(+ 1)	0.5(-1)	72(+ 1)	30(+ 1)	0.175	0.1941	-0.0191
85	30(-1)	5(-1)	2.5(+ 1)	0.5(-1)	72(+ 1)	10(-1)	0.202	0.1922	0.0098
86	30(-1)	9(+ 1)	0.5(-1)	2.5(+ 1)	24(-1)	10(-1)	0.274	0.2449	0.0291

$$\begin{aligned}Y\:&=\:\--\:0.045\--0.023\:X_1+0.102\:X_2\:\--\:0.124\:X_3\\&+0.016\:X_4+0.006\:X_5+0.026\:X_6\:\--\:0.001\:X_1X_3\:\-\\&\:-0.001\:X_2X_4\:\--\:0.005\:X_3X_4\:\--\:0.006\:\left(X_2\right)2\:\\&+\:0.054\:\left(X_3\right)2\:+\:0.013\:\left(X_4\right)2\:\-\:0.001\left(X_6\right)2.\end{aligned}$$

Where Y represents the response or pigment yield, and X_1_, X_2_, X_3_, X_4_, X_5_, and X_6_ are temperature, pH, mannitol percentage, peptone%, incubation time, and ZnCl2, respectively.

### Model validation

The validity of the proposed approach was evaluated by anticipating the level of STX production in each trial of the matrix for *S. aureus* A2. The results, as presented in Table 3, indicated that the highest recorded STX production (OD456 = 0.328) closely matched the projected value (OD456 = 0.3089) in run 50. Tables 4 and 5 demonstrate the outcomes of the analysis of variance for STX production. The model was highly significant with a F value of 15.01, as evidenced by the results of Fisher’s F test, which indicated a very low probability value (P model > F = 0.01). The experiments were more precise and reliable as the value of ‘Prob > F’ was less than 0.0001 and the coefficient of variation was comparatively lower (8.09%). All factors had a substantial impact on the STX production statistics, and the findings exhibited an excellent correlation between the anticipated and actual values. A p-value of less than 0.0500 is required to indicate that the model terms are statistically significant. The following model terms were significant in the case of: A, B, C, D, E, F, AB, AC, AF, BE, CD, CE, DE, EF, B², C², E², F². On the other hand, values greater than 0.1000 indicated that the model terms lack significance. The insignificant Lack of Fit is less than the pure error, indicating that the model adequately fits the data. The adjusted R^2^ of 0.8166 is in reasonable agreement with the predicted R^2^ of 0.6958, where discrepancy was less than 0.2. An acceptable level of precision is achieved by measuring the signal-to-noise ratio. There is a preference for a ratio that exceeds 4. An adequate signal is indicated by the obtained ratio of 16.5135, emphasizing the success of the design.

**Table 4:** Analysis of variance (ANOVA) of the response surface quadratic model CCD on STX production by *S. aureus* A2 isolate

Source	Sum of squares	Degrees of freedom	Mean square	F-value	*p*-Value Prob > F	
Model	0.1294	27	0.0048	15.01	< 0.0001	significant
A- temperature	0.0027	1	0.0027	8.62	0.0048	
B- pH	0.0075	1	0.0075	23.46	< 0.0001	
C- mannitol	0.0018	1	0.0018	5.78	0.0194	
D-peptone	0.0472	1	0.0472	147.89	< 0.0001	
E-incubation time	0.0039	1	0.0039	12.30	0.0009	
F-ZnCl2	0.0020	1	0.0020	6.12	0.0163	
AB	0.0031	1	0.0031	9.83	0.0027	
AC	0.0029	1	0.0029	8.97	0.0040	
AD	0.0005	1	0.0005	1.66	0.2031	
AE	0.0002	1	0.0002	0.7050	0.4046	
AF	0.0019	1	0.0019	5.86	0.0186	
BC	0.0001	1	0.0001	0.3454	0.5590	
BD	0.0006	1	0.0006	1.80	0.1844	
BE	0.0023	1	0.0023	7.22	0.0094	
BF	0.0001	1	0.0001	0.2399	0.6261	
CD	0.0017	1	0.0017	5.40	0.0237	
CE	0.0014	1	0.0014	4.41	0.0402	
CF	0.0006	1	0.0006	1.77	0.1889	
DE	0.0079	1	0.0079	24.82	< 0.0001	
DF	0.0005	1	0.0005	1.48	0.2284	
EF	0.0038	1	0.0038	11.95	0.0010	
A²	0.0009	1	0.0009	2.79	0.1000	
B²	0.0014	1	0.0014	4.37	0.0409	
C²	0.0072	1	0.0072	22.49	< 0.0001	
D²	0.0005	1	0.0005	1.43	0.2366	
E²	0.0039	1	0.0039	12.08	0.0010	
F²	0.0086	1	0.0086	27.10	< 0.0001	
Residual	0.0185	58	0.0003			
Lack of Fit	0.0185	49	0.0004			
Pure Error	0.0000	9	0.0000			
Cor Total	0.1479	85				

**Table 5:** Regression values of CCD

Std. Dev.	0.0179	*R*²	0.8748
Mean	0.2208	Adjusted R²	0.8166
C.V. %	8.09	Predicted R²	0.6958
		Adeq Precision	16.5135

C.V. Coefficient of variation

R^2^. Squared regression coefficient

### Interactive effects of the variables

**Fig. 5:**
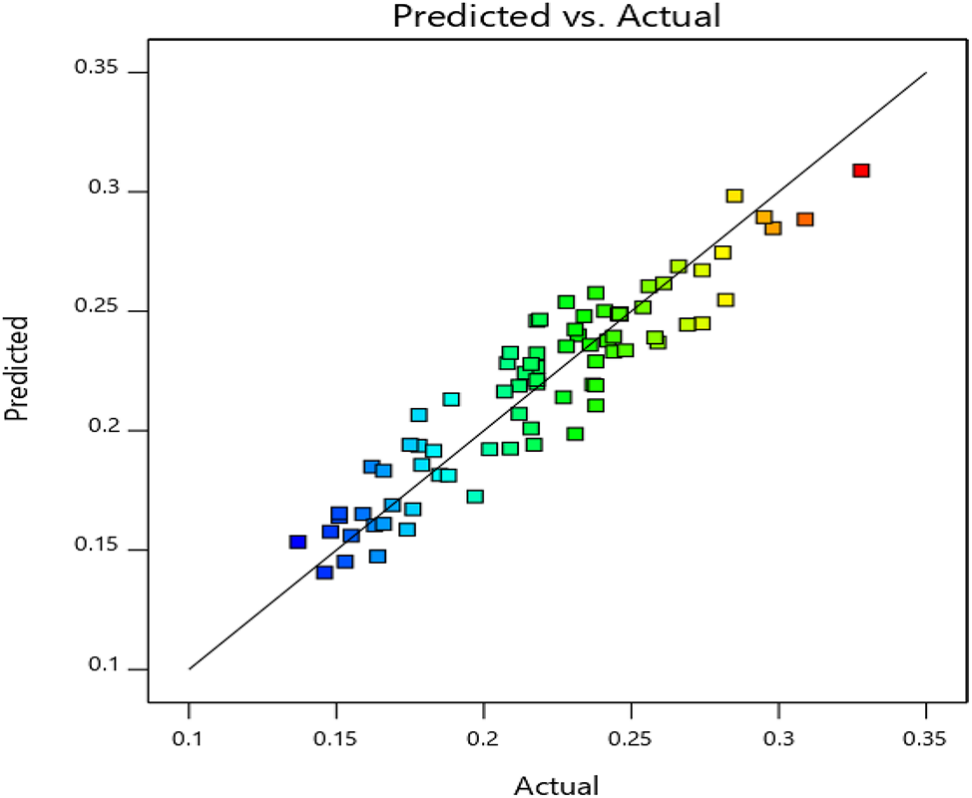
Normal probability plot for STX production

**Fig. 6:**
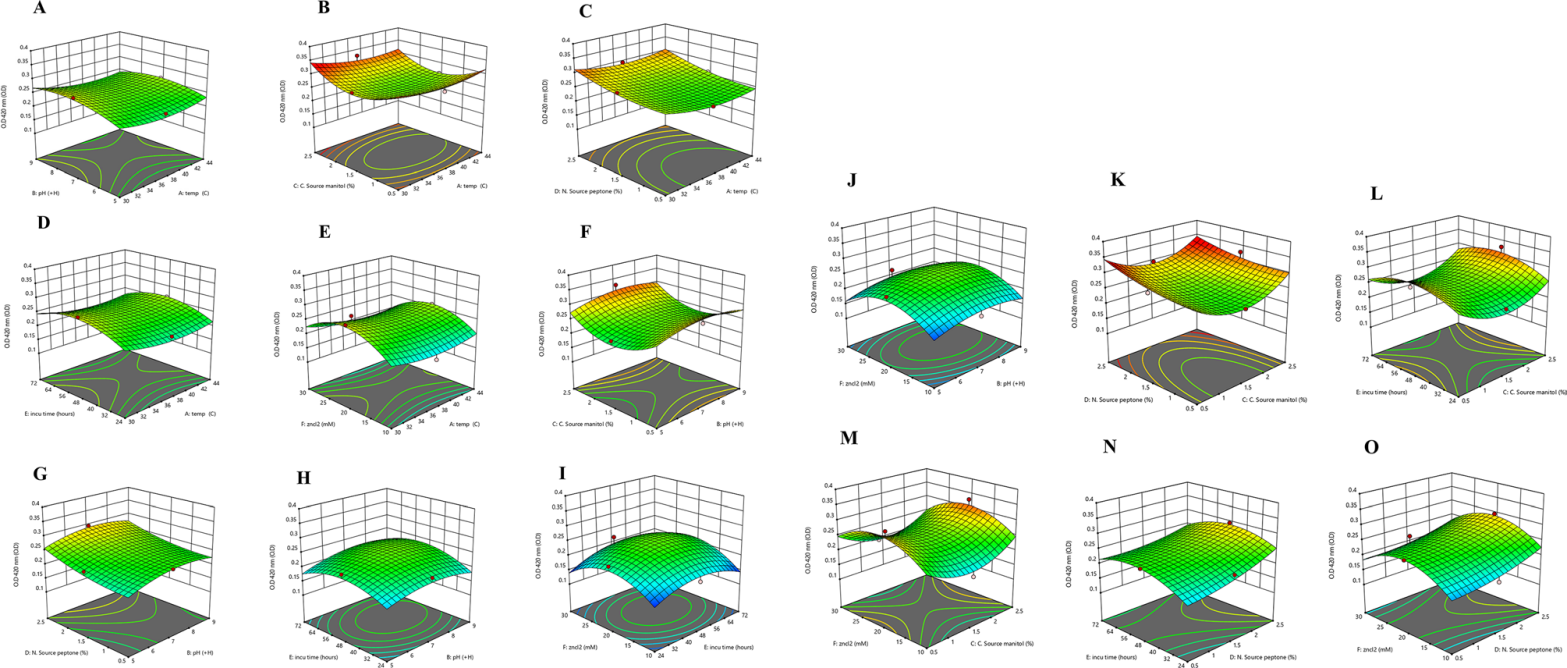
3D response surface plots of the interactive effects between variables (**A)** temp, pH; (**B**) temp, mannitol; (**C)** temp, peptone; (**D**) temp, incubation; (**E**) temp, ZnCl2; (**F**) pH, mannitol; (**G**) pH, peptone; (**H**) pH, incubation; **(I**) incubation, ZnCl2; (**J**) ZnCl2, pH; (**K)** peptone, mannitol; **(L**) mannitol, incubation; (**M**) mannitol, ZnCl2; (**N**) peptone, incubation; and (**O)** peptone, ZnCl2

The significant interaction effects of various variables on STX production were examined using the three-dimensional profiles of multiple nonlinear regression models depicted in Figs. 5 and 6. The residual distributions were analyzed using the normal probability plot (Fig. 5), which revealed a straight line, indicating that the errors were equitably distributed, which supports the adequacy of the model. To accurately identify the ideal level of each factor for the maximum response and illustrate how various factors interact, 3D response surface curves were developed. Our results illustrated the interaction effects of the factors in maximizing the yield of STX between any two independent variables, whereas the remaining independent variable is archived at its zero level (Fig. 6).

### Extraction and purification of staphyloxanthin

**Fig. 7:**
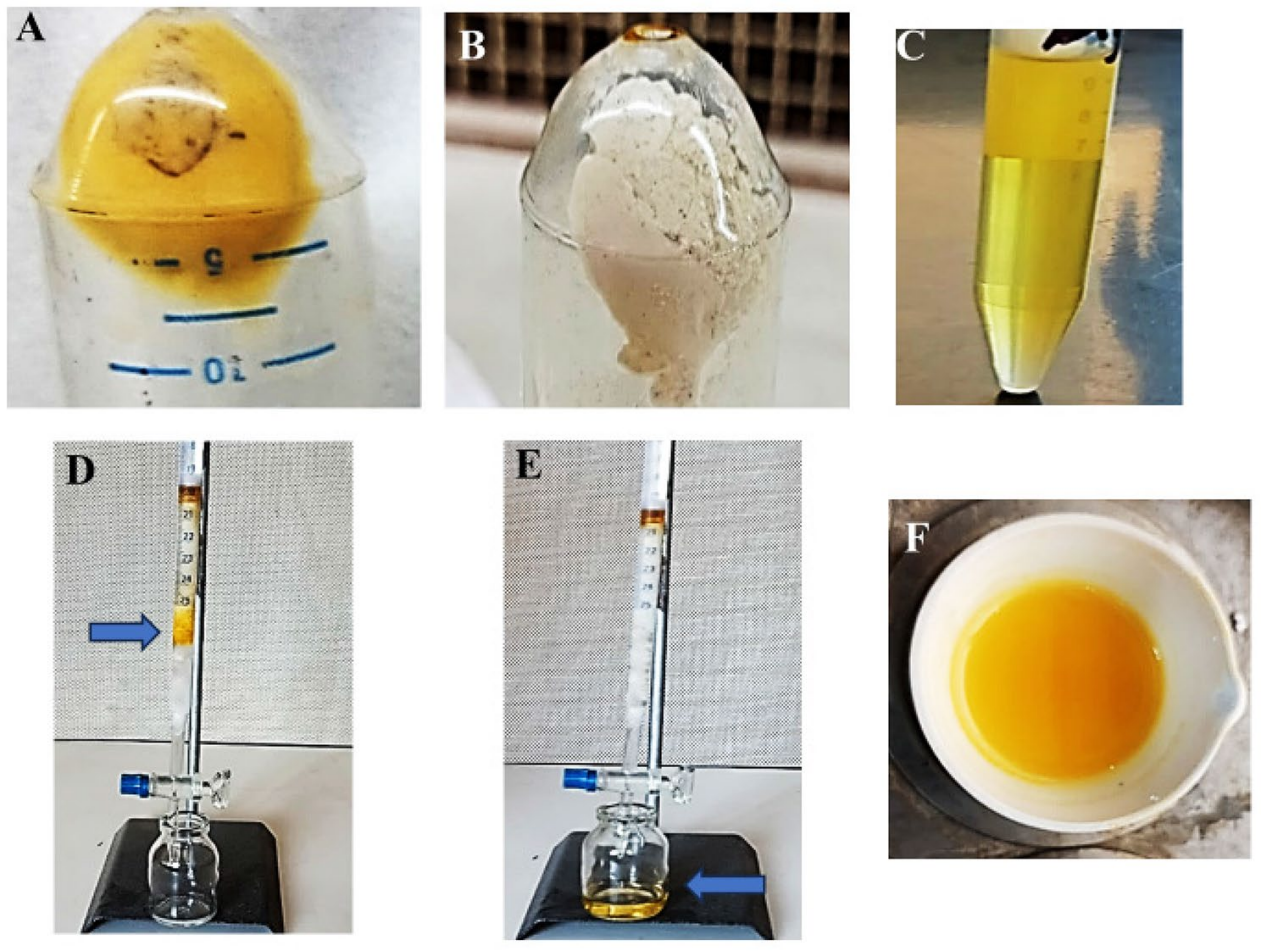
Extraction and purification of STX pigment. (**A**) pigmented cell pellets; (**B**) cell pellets lost their color after exhaustion with methanol; (**C)** colored supernatant collected after centrifugation; (**D**) silica gel column chromatography showing golden-yellow band (arrowed); (**E**) collection of the colored fraction after elution (arrowed); **(F)** evaporation of the mobile phase and collecting the pigment powder

STX pigment was produced during the growth of *S. aureus* A2 strain in a 250-mL Erlenmeyer flask containing BHI broth with optimal levels of growth parameters, including pH 7, 2.5% mannitol, 1.5% peptone, and 20 mM ZnCl2, and incubated at 37 °C with shaking (200 rpm) for 48 h. The pigment was extracted with absolute methanol, as shown in Fig. 7. After the centrifugation process, silica gel column chromatography was used to purify the colored supernatant. The fractionated mobile phase was used to elute the sample until the characteristic golden-yellow band appeared and was successfully eluted, as shown in Fig. 7. The golden-yellow-colored eluted fractions were collected, and the mobile phase was evaporated in a porcelain dish at 40 °C. Finally, the purified powder was then stored in darkness at 4 °C for further characterization and biological evaluation.

### Chemical structure validation of the purified pigment

**Fig. 8:**
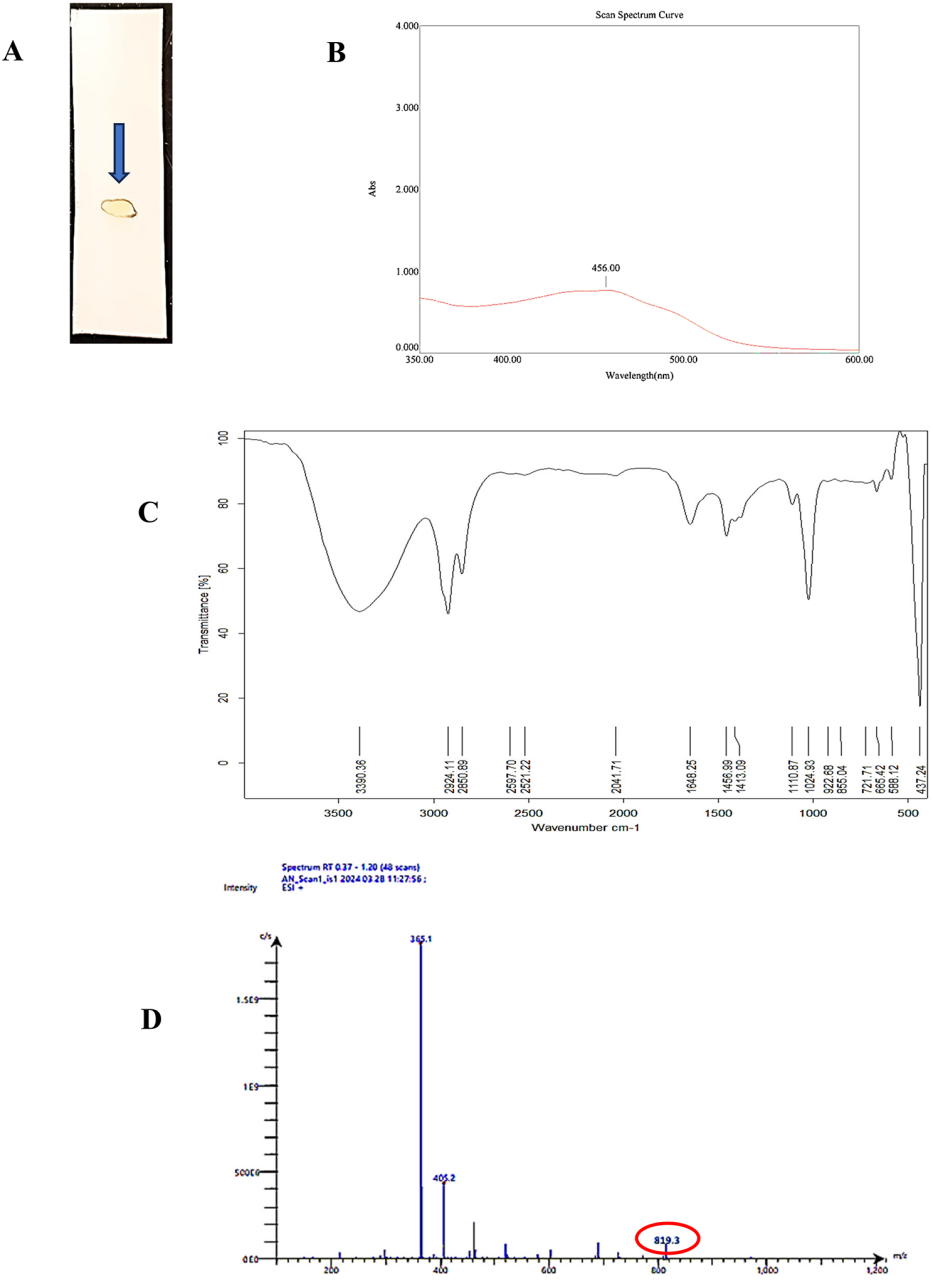
Characterization of the purified pigment. (**A**) TLC plate showing characteristic yellow spot; (**B)** UV–VIS spectrum; (**C**) FT-IR spectrum; and (**D**) TLC-ESI–MS/MS chromatogram

The purified STX pigment was characterized, and the putative identity was confirmed by TLC, UV–VIS, FT-IR, and TLC-ESI-MS/MS analysis. The TLC plate displayed a significant golden-yellow colored spot with a retardation factor of 0.38 that was identical to previously reported STX data [26, 42], confirming the presence of the pigment, as shown in Fig. 8A. The maximum absorption wavelength of the UV–vis spectrum for purified STX was 456 nm (Fig. 8B). The FTIR absorption spectra (Fig. 8C) indicated the presence of C = O groups at a frequency of 1648 cm^–1^ and OH groups at a frequency of 3390 cm^–1^. The peaks observed at 2924 cm^− 1^ were allocated to the aliphatic CH stretch, while the peaks at 1110–1024 cm^–1^ correspond to the stretch of the ester group. In addition, a C-C stretch at a frequency of 1456 cm^− 1^ was observed. The observed functional groups are a result of the presence of certain components such as fatty acid ester, β–D-glucopyranose, and methyltetracosane in the STX structure. Additionally, the TLC-ESI-MS/MS analysis was used to determine the molecular mass of the hypothetical STX. The TLC-ESI-MS/MS study determined that the molecular mass of STX was 819.33 m/z for the precursor ion [M + H]^+^. Furthermore, the MS/MS results confirmed the molecular structure by providing fragment ions at 365.1 and 405.2 m/z (Fig. 8D), which were in agreement with the fragmentation pattern found in the published data [43]. Therefore, the purified sample shared the same molecular and chemical structure of STX, as well as the same spectral features, according to the combined findings of UV–VIS, FTIR, and TLC-ESI-MS/MS.

### Safety profile of purified staphyloxanthin pigment

**Fig. 9:**
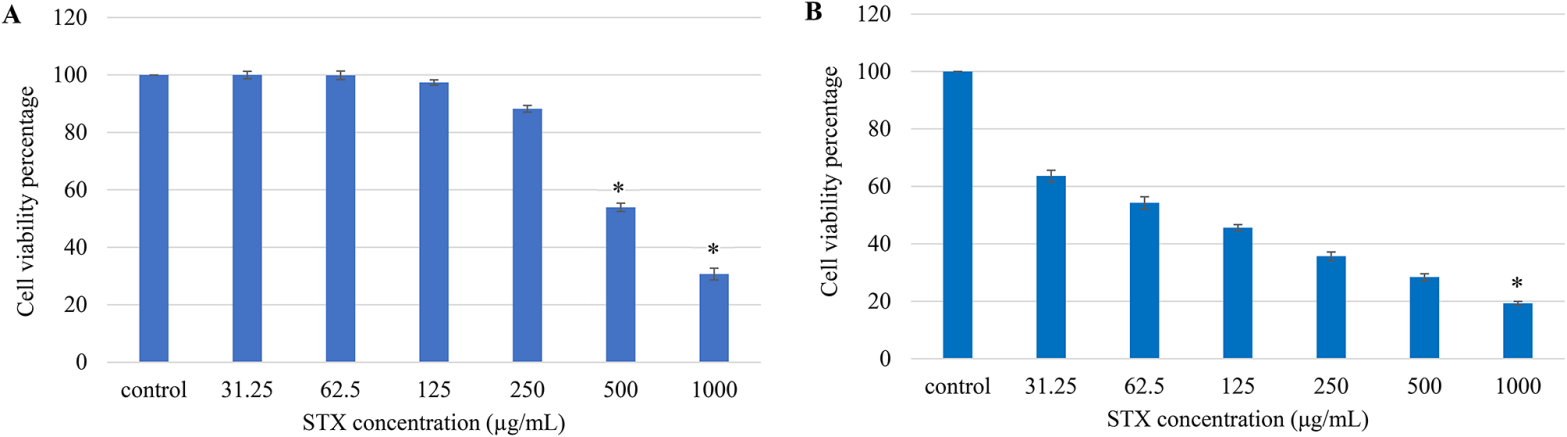
Determination of cytotoxic activity of STX pigment. (**A**) cytotoxicity on normal Vero cells and (**B**) cytotoxicity on A549 lung cancer cells. Data represented as mean ± SD (*n* = 3). The asterisks denote statistical significance at *P* < 0.05 compared to control (ANOVA and Tukey’s post-test)

It was necessary to determine the sub-toxic concentrations of STX in order to use it in subsequent investigations. To accomplish the aforementioned, normal Vero cells were exposed to increasing levels of STX, and the MTT assay was performed to measure the cytotoxicity. It was noticed that the IC50 of STX on Vero cells was 523 µg/mL, as shown in Fig. 9A. Notably, the survival rate of approximately 100% was maintained until it surpassed a concentration of 125 µg/ml. The results demonstrated that normal Vero cells were able to withstand STX, indicating that STX can be safely used at concentrations around 125 µg/ml.

### Cytotoxic activity against non-small cell lung cancer (NSCLC)

The MTT assay was implemented to assess the impact of STX on A549 lung carcinoma cell line. The anticancer scrutiny revealed that the STX pigment exhibited significant cytotoxic activity (*P* < 0.05) against A549 cells compared to normal Vero cells. This is expected, as tumor cells typically have a cellular growth rate around 40 times higher than that of normal cells. The results displayed in Fig. 9B showed that the IC50 of STX on A549 was 57.3 µg/mL. Thus, the selectivity factor of STX towards A549 cells, normalized to normal Vero cells, was approximately 10 folds.

### Investigation of necrosis and apoptosis induction using confocal laser scanning microscopy (CLSM)

**Fig. 10:**
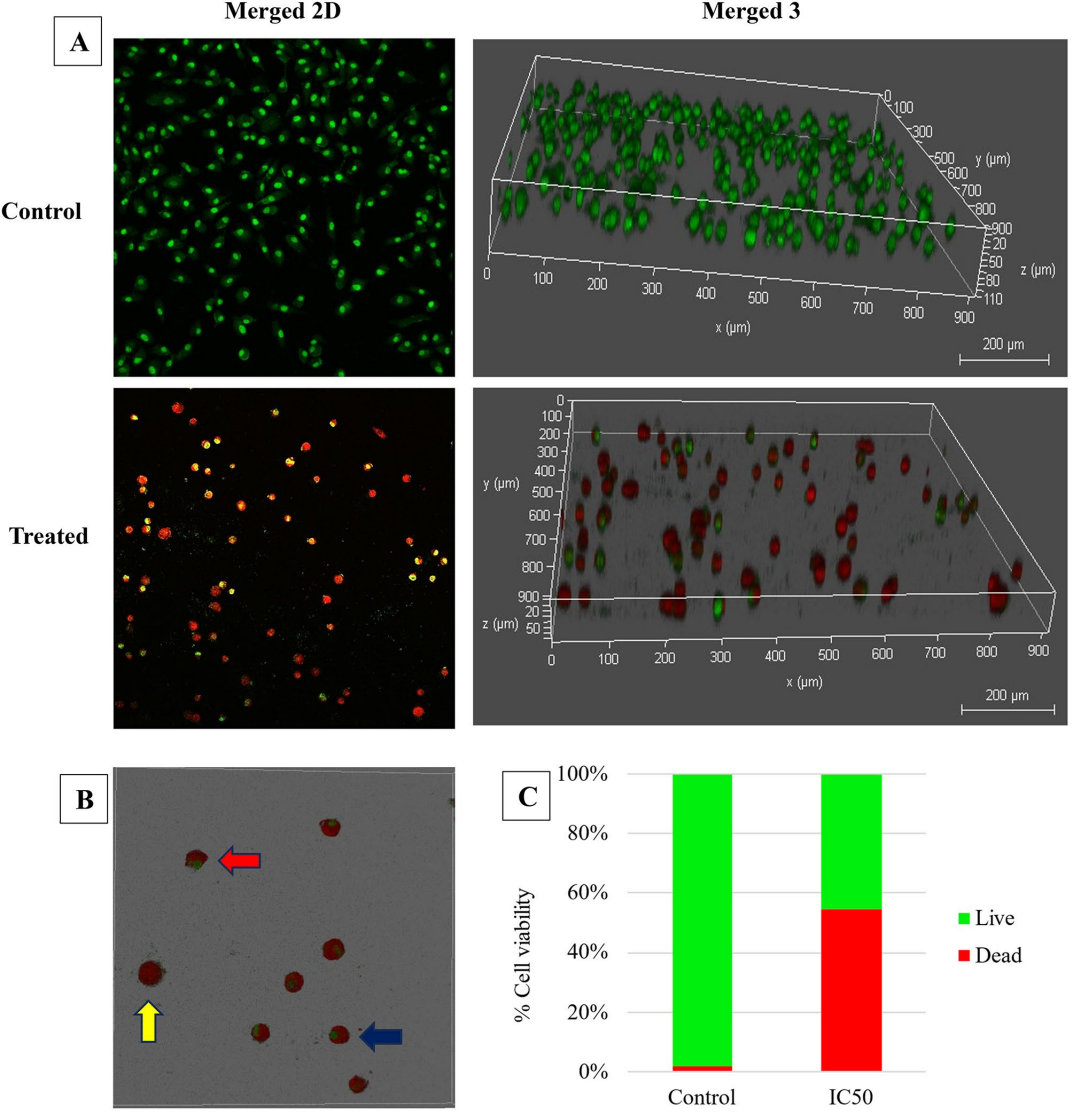
Fluorescent micrographs (10× magnification) of A549 cells double-stained with acridine orange and propidium iodide (AO/PI) after STX treatment. (**A**) Untreated viable A549 cells display green fluorescence, while red fluorescence emission signifies the presence of necrotic and apoptotic cells post-treatment. (**B**) Higher magnification (20×) of treated cells exhibiting membrane blebbing (yellow arrow), late apoptotic cells (blue arrow), and necrotic cells (red arrow). (**C**) Quantitative statistic depicts the percentage of dead and viable cells before and after treatment with STX at concentration of IC50

The visualization of apoptosis and necrosis in both untreated and pigment-treated A549 cells at IC50 was achieved through confocal microscopy, employing a double staining technique with acridine orange and propidium iodide (AO/PI). After 24-h incubation, untreated A549 cells were viable, displaying distinct, well-defined forms and glowing green fluorescent light, as shown in (Fig. 10A). Conversely, A549 cells treated with STX emitted red fluorescence light as a result of the apoptotic and necrotic processes. Treatment of the cancer cells with IC50 of STX resulted in the morphological alterations of the apoptotic cells, including membrane blebbing, cell shrinkage, and appearance of apoptotic bodies, as depicted in Fig. 10B. Moreover, the statistically substantial reduction in fluorescence means intensity and 3-dimensional analysis demonstrated that the thickness of monolayer cells was significantly reduced by 54% following treatment (Fig. 10A and C).

### Annexin V-FITC apoptosis assay

**Fig. 11:**
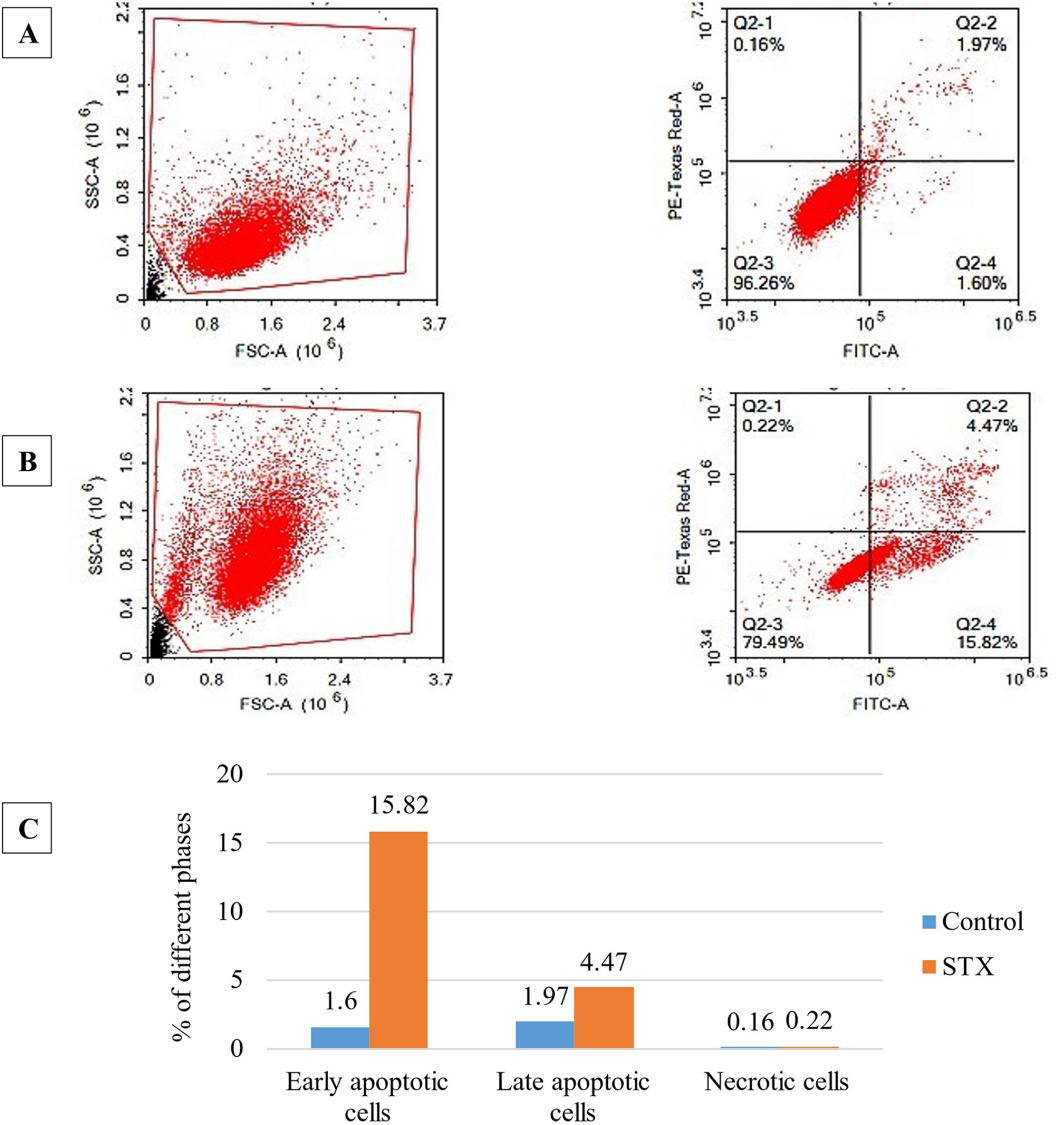
Determination of apoptosis assay using flow cytometry. The Annexin V-FITC/PI-positive staining of (**A**) control and (**B**) A549 cells treated with STX. The four quadrants were labeled as follows: Q1 for necrotic, Q2 for late apoptotic, Q3 for normal intact cells, and Q4 for early apoptotic cells. (**C**) Bar chart presentation of apoptotic and necrotic cells percentages

Apoptosis induction is the most critical pathway that lets chemotherapeutic agents exterminate cancer cells. Annexin V-FITC apoptosis assay was employed to ascertain whether the suppression of A549 cell proliferation following STX treatment is linked to the initiation of apoptosis and/or necrosis. Using the ACEA Novocyte™ flow cytometer to perform cytometric analysis, the A549 cells, both untreated and treated, were subjected to dual-staining with Annexin V-FITC and PI stains. The results displayed in Fig. 11 (lower-right quadrant of the cytogram) showed that the early apoptosis ratio exhibited a significant increase to 15.82%, which is approximately 10-fold increase, compared to the value of 1.6% in the control cells, while the late apoptosis ratio (upper-right quadrant of the cytogram) increased to 4.47% following treatment, compared to 1.97% in the control cells (2-fold increase). Furthermore, the necrotic cells (upper-left quadrant of the cytogram) slightly increased from 0.16 to 0.22%. These findings suggested that the cytotoxic effects of the STX were mostly triggered by the apoptotic mechanism rather than the necrotic pathway.

### Cell cycle arrest analysis

**Fig. 12:**
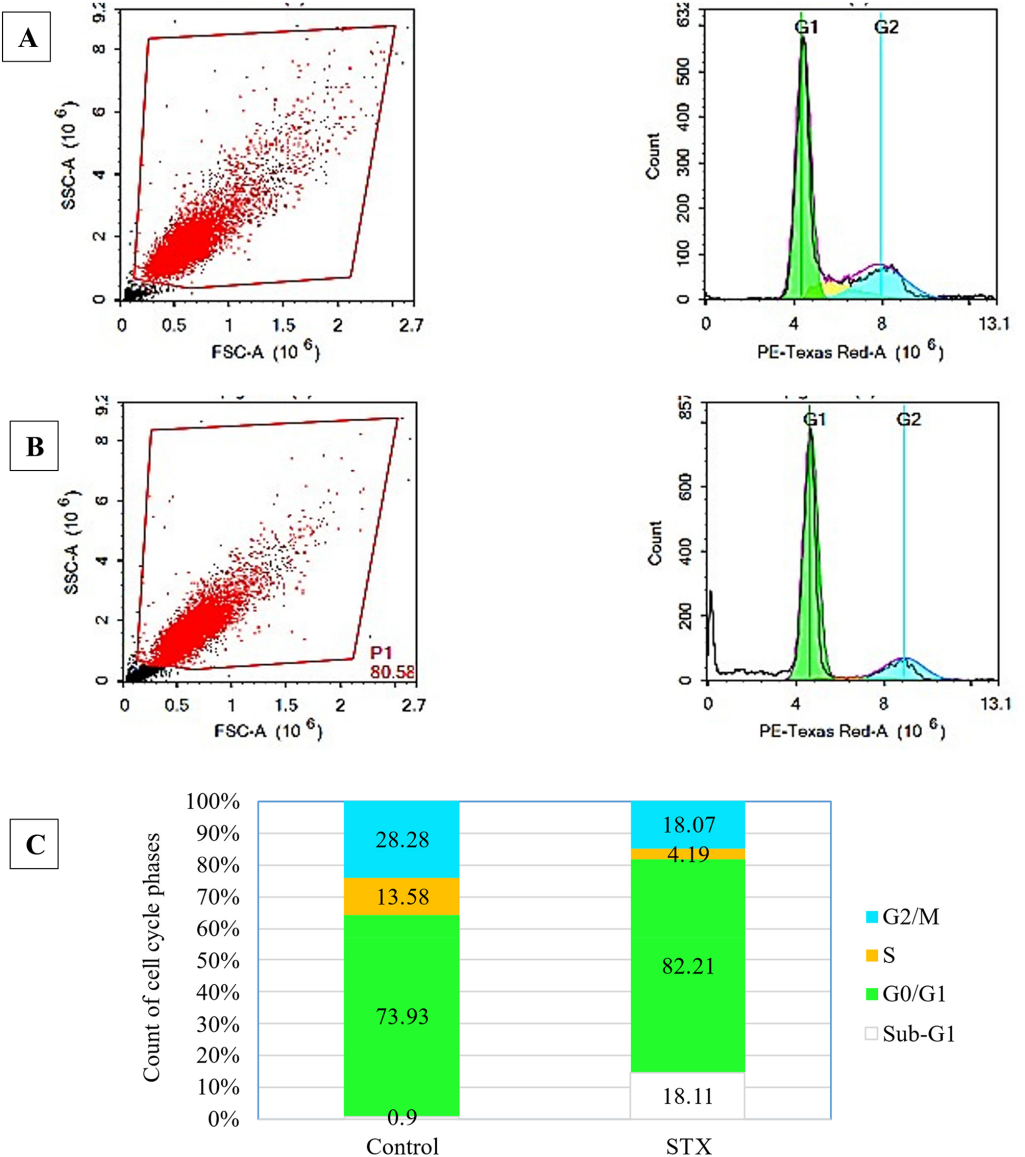
Determination of cell cycle arrest using flow cytometry. The distribution of cell phases (sub-G1, G0/G1, S, and G2/M) of (**A**) control and (**B**) 24-h STX treated A549 cells were represented. (**C**) Bar chart presentation of cell phases distribution

**Fig. 13:**
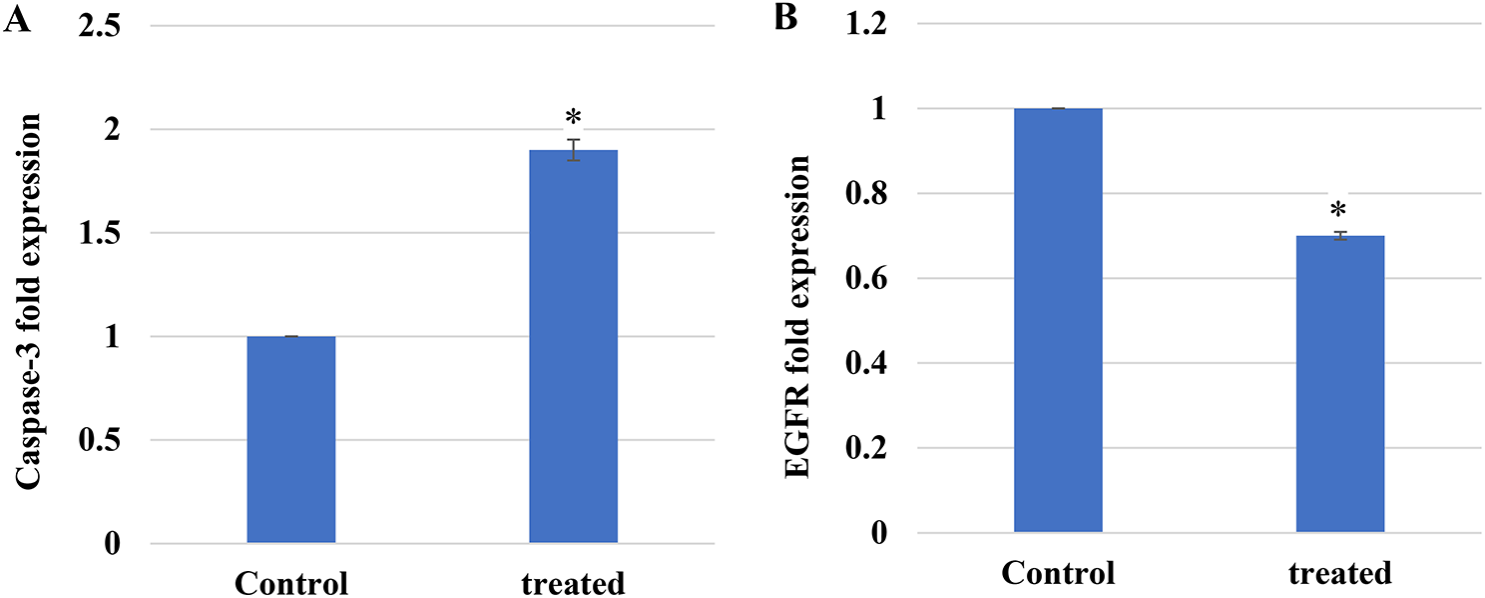
The STX impact on gene expression level of caspase-3 (**A**) and EGFR (**B**) in A549 lung cancer cells. Data represented as mean ± SD (*n* = 2). The asterisks denote statistical significance at *P* < 0.05 compared to control (Student’s ttest)

The cell cycle is a sequence of successive events that culminate in DNA replication and cell division. To evaluate the effect of STX pigment on the progression of cell cycle in A 549, the cells were subjected to cell cycle analysis. To accurately define the mode of cell death triggered by the purified pigment, the cells were stained with propidium iodide (PI), and the DNA content was quantified using the ACEA Novocyte™ flow cytometer. Treatment with STX resulted in a remarkable increase in pre-G1 phase (18.11%), which was approximately 21 folds more than the initial value 0.9% of in the control cells, as shown in Fig. 12. Additionally, there was an increase in the percentage of cells in the G0/G1 phase, with a percentage of 82.21%, compared to a baseline of 73.93% in the control cells. On the contrary, there was a decrease in the overall percentage of S-phase cells to 4.19%, compared to 13.58% in the control population (3-fold decrease). Moreover, STX treatment resulted in an overall decrease of the cell proportion at the G2/M phase to 18.07%, compared to 28.28% in the control cells. Based on our results observed in Fig. 13, we concluded that STX cause programmed cell death (apoptosis) in NSCLC, A549 cells, by disrupting the cell cycle at both the pre-G1 phase and the G0/G1 phase.

### Gene expression assessment

Caspase-3 is essential for apoptosis and considered a therapeutic target for anticancer drugs. A549 cells treated with STX were subjected to real-time PCR utilizing caspase-3 specific primers. As seen in Fig. 13A, treated A549 cells exhibited significantly (*P* < 0.05) higher expression of caspase-3 than the untreated cells. Furthermore, the epidermal growth factor receptor plays a crucial role in NSC lung cancer, and its activation promotes tumor growth, invasion, and metastasis. The STX impact on EGFR expression in A549 cells was assessed using real time PCR. According to the findings in Fig. 13B, STX treatment significantly (*P* < 0.05) reduced EGFR expression by almost 1.5-fold. Our findings indicated that EGFR inhibition and caspase-3 activation were necessary for STX-induced apoptosis and cell growth in the NSCLC cell line (A549).

### Molecular Docking analysis

**Fig. 14:**
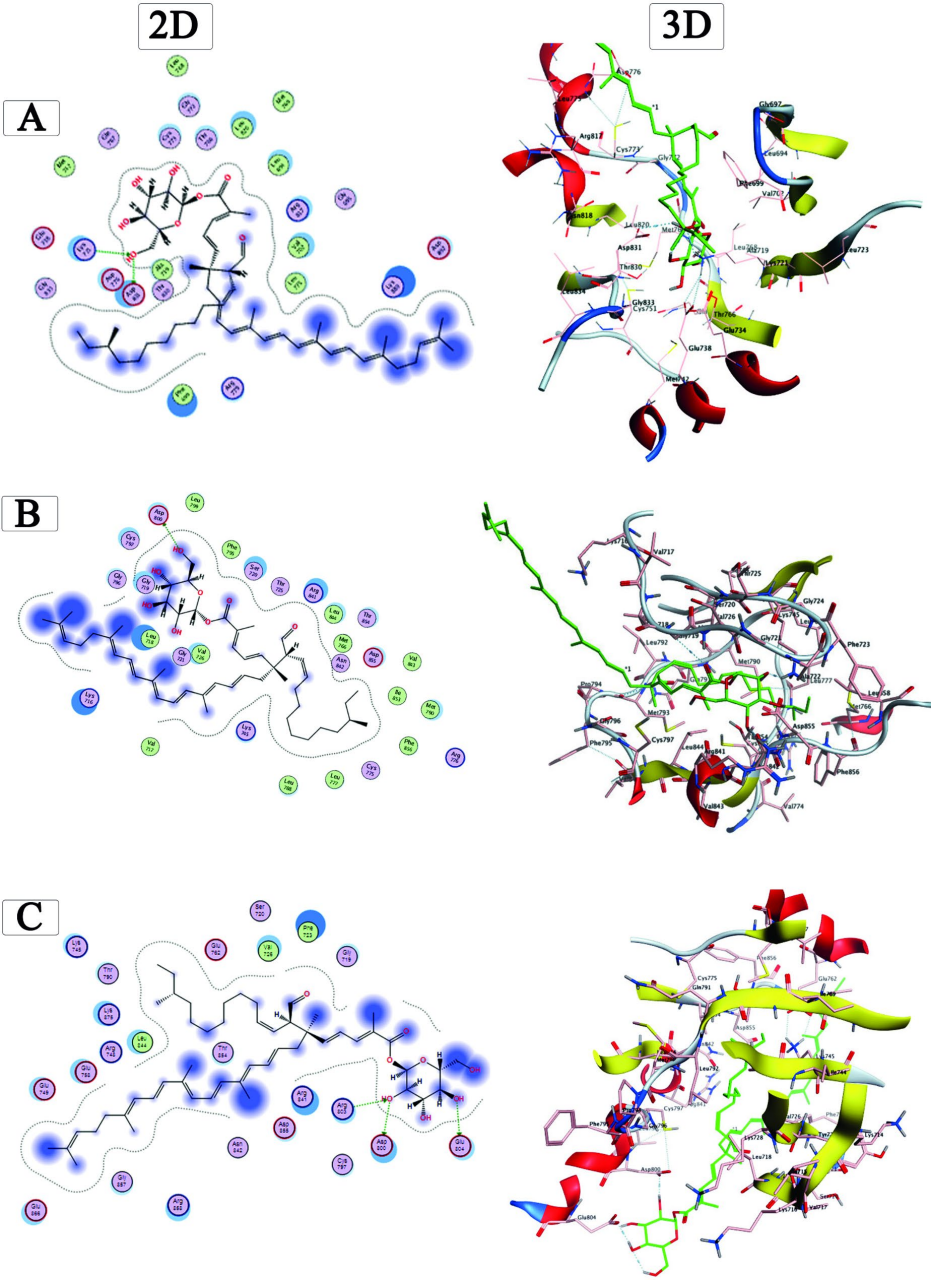
Molecular docking analysis showing 2D and 3D binding modes of STX residue docked and minimized in the EGFR binding pocket using the following PDB codes. (**A)** EGFR^WT^ (PDB code: 1M17), (**B**) EGFR^T790M^ (PDB code: 2JIV), and (**C)** EGFR^L858R^ (PDB code: 4LQM)

Using the “molecular operating environment (MOE) version 2019.0102,” a molecular docking study was conducted to acquire an improved comprehension of the manner in which the purified STX pigment binds to the ATP-binding pocket in the crystal structure of the wild type enzyme EGFR^WT^ (PDB code: 1M17). The active site of the EGFR enzyme is primarily targeted by first generation EGFR-tyrosine kinase inhibitors (TKIs). Results of docking of STX into the ATP-binding pocket of EGFR^WT^ (Score= -10.20 kcal/mol) illustrated that STX interacts with EGFR^WT^ through hydrogen bonding interactions, formed with Lys721 and Asp831. The docking analysis was further extended to investigate the interactions between the STX and the ATP-binding pocket in the crystal structures of the mutant types EGFR^T790M^ (PDB code: 2JIV) and EGFR^L858R^ (PDB code: 4LQM) that were targeted by second and third generation EGFR TK inhibitors, respectively [44, 45]. Regarding EGFR^T790M^, docking results of STX exposed many hydrogen bonding interactions with Lys745, Met790, Arg841, and Asn842 with a binding score of -11.10 kcal/mol. Docking of STX into EGFR^L858R^ (Score= -9.13 kcal/mol) revealed hydrogen bond interactions with Asp800, Arg803, and Glu804. All interactions and docking scores are displayed in details in Fig. 14. The analysis of binding scores revealed that upon docking into the ATP-binding pocket of EGFR, STX exhibited a greater binding affinity to the 4LQM protein compared to the 1M17 and 2JIV proteins. In conclusion, the docking results strongly suggest that STX effectively displaces the charged ATP ligand of both wild-type and mutant EGFR.

## Discussion

Recently, there has been a notable surge in the global interest in natural pigments, recognized as more secure alternatives to their synthetic counterparts. Particularly, microbial pigments are preferred owing to their easy and quick extraction and scaling up [4]. Microbial fermentation provides an array of beneficial products. Bacterial pigments such as indigoidine, melanin, violacein, pyoverdine, rhodopsins, prodigiosin, and carotenoids are employed in the food, cosmetics, pharmaceutical, and textile sectors because of their safety [32]. The advancement in microbial pigment research can be attributed to the diverse and significant biological purposes of carotenoid and apocarotenoid pigments across various living forms. The industry is fascinated by applying carotenoid pigments for human consumption since they offer safety and biodegradability, particularly juxtaposed with synthetic pigments [46, 47]. The research scope of the biological attributes of apocarotenoid pigments, especially STX pigment, is preliminary and still in need. Hence, this study highlights the determinants influencing STX production, the enhancement of both STX productivity and cellular proliferation, and the exploration of its anticancer activity.

In this study, 59 isolates of *S. aureus* were screened and assayed for STX production. The highest STX-producer strain, *S. aureus* A2, was selected for optimization of the fermentation conditions to maximize the pigment production. Regardless of the process used to produce metabolites, both physicochemical and nutritional key process variables differ from one organism to another. In a particular bioprocess, the process variables necessary for the metabolite production may be regulated by the physiological and metabolic traits of the organism involved [48, 49]. The composition of the medium influences both the accumulation of metabolic products and cellular growth [50]. Therefore, a vital aspect of bioprocess development is the assessment and evaluation of nutritional requirements. According to our OFAT optimization results, pigmentation appeared to be controlled by both the specific type of medium and the incubation period. The pigmentation was less noticeable on low-nutrient media, such as glucose broth and peptone water, and more prominent on nutrient-rich BHI medium. These findings were congruent with prior studies indicating that bacterial pigments production augmented with elevated carbon and nitrogen supplies in the fermentation media [24, 51]. The integration of minerals within a microbial ecosystem enhances their developmental capabilities by offering essential physical and chemical safeguards, as well as vital nutrition and energy sources [52]. Enzymatic activity is contingent upon micronutrients. In numerous instances, they participate in metabolic regulation by serving as cofactors or prosthetic groups for critical metabolic enzymes [50]. In the present study, incorporation of ZnCl_2_ as micronutrient in the medium composition showed a positive impact on STX production. In accordance with prior research, these results demonstrated that the production of metabolic products was enhanced by the presence of mineral and vitamin supplies in the fermentation media [22, 50]. Moreover, pigmentation production peaked at 72 h and reached its nadir at 96 h under our experimental conditions. Extending the incubation period may enhance pigmentation; however, it concurrently leads to diminished pigment synthesis after 72 h. This is due to the deterioration of the cells, resulting in a reduced capacity to assimilate nutrients. The reduction of essential nutrients and the initiation of the organism’s mortality phase may both contribute to the observed outcomes [22]. In addition, the pigment degradation due to interactions with various components in the medium may be another possible cause that resulted to a reduction in pigment production [22, 53, 54].

It has been documented previously that environmental stress factors, such as pH, temperature, and salt concentrations, can have a consequence on the pigmentation of bacterial species [55]. This study observed that the optimal stress factors for STX production included a temperature of 37 °C and a pH of 7. It is noteworthy that a prior report concerning optimal carotenoid pigment production in *Rhodotorula slooffiae* at 37 °C indicated a gradual decline in pigmentation with increasing temperature over 40 °C [53]. This may be attributed to the fact that elevated temperatures impede bacterial proliferation, as temperature influences both bacterial metabolism and cellular viability. The production of the pigment diminishes with an increase in temperature. The findings revealed that the peak occurred at 37 °C, suggesting a direct correlation between pigment production and biomass, thereby indicating that pigment production is contingent upon growth [22, 56, 57]. The alteration in the microstructure of the growth medium is also regarded as an environmental stressor that impacts the interplay between various environmental factors, such as pH, water activity, and temperature, on microbial growth [58]. Numerous investigations have demonstrated that, in contrast to planktonic cells, immobilized cells have distinct metabolic activities and a slower growth rate [59, 60]. Another study reported that there was a noticeable difference in the growth rate of *Bacillus cereus* cells cultured in broth and those immobilized on gelatin gel [61]. In a similar vein, researchers have shown that *Aspergillus carbonarious* trapped in gel produces less ochratoxin A compared to when grown in liquid media, in addition to a slower growth rate (measured as an increase in biomass) [62].

The current study employed RSM optimization to assess the fundamental and interactive effects of various fermentation parameters on STX production. The findings indicated the highest enhancement in STX pigmentation under specific culture conditions, which included a temperature of 37 °C, a pH of 5, an incubation duration of 48 h, a mannitol concentration of 2.5%, a peptone concentration of 1.5%, and a ZnCl2 concentration of 20 mM. The use of RSM is gaining prominence for its ability to efficiently collect optimal conditions for multifactorial experiments. These findings are in accordance with prior research on the utilization of RSM to optimize pigment production culture conditions. Hegazy et al. utilized RSM to refine the culture conditions for the notably productive *Micrococcus luteus* (ATCC 9341) strain, resulting in optimal carotenoid production. This was accomplished by adjusting the culture parameters to 3% whey, an agitation speed of 175 rpm, a pH of 7, and a 7.5% inoculum size at a temperature of 32.5 °C [15]. El-Zawawy et al. also employed RSM to enhance melanin production from *Streptomyces djakartensis* NSS-3 cultivated on low-cost agricultural wastes [63]. Moreover, Prabhu et al.. employed RSM to enhance and optimize the extraction of Betalain pigments from the promising *Beta vulgaris*, attaining a peak extraction time of 21.14 min, a solid-to-liquid ratio of 21.61 mg/mL, and a temperature of 52.98 °C [64]. RSM demonstrates greater accuracy compared to traditional methods for optimizing pigment production. The model’s precision is verified, and the optimal operating conditions are forecasted utilizing the evaluation parameters *P*-value and R^2^ [65]. The significance of the variables is considered when the *P*-value is less than 0.05. R^2^ is a metric that quantifies the extent to which the model explains the variation around the mean. The quality of fit of the quadratic model equation is denoted by R^2^ [65]. The strong correlation between the fitted model and the experimental data is indicated by the close proximity of the predicted R^2^ value (0.6958) to the adjusted R^2^ value (0.8166), underscoring the success of the design [66, 67].

The lack of authentic STX impeded the HPLC analysis, which serves as a crucial confirmation for the identification process. Therefore, the structure identity of STX was chemically validated using TLC, UV–Vis, FTIR, and TLC-ESI-MS/MS. The putative STX demonstrated consistent structural identity, functional groups, and molecular fragmentation patterns coincident with the reported data [26, 42, 43]. Furthermore, the cytotoxic effect of STX was examined using a normal Vero cell line to determine biosafety for prospective biotechnological and clinical uses. It is noteworthy that a significant level of cell survival was observed even with the administration of 250 µg/mL STX treatment, with more than 90% of viability maintained, thereby affirming a wide margin of tolerability and a minimal risk of toxicity. The minimal toxicity enhances the practicality of safe application for further investigations, supported by elevated IC50 values. In addition, the anticancer activity of STX against NSCLC was examined. The investigated inhibitory activity of extracted pigment on cell viability demonstrated that the IC50 value for A549 cells was 57.3 µg/mL. Based on the standards set forth by the Geran protocol and the National Cancer Institute, extracts exhibiting an IC50 range of 21 to 200 µg/mL are deemed significantly cytotoxic [68, 69]. This substantiates the assumption that the pigment holds potential as an anticancer agent, capable of eradicating cancer cells at minimal concentrations while preserving the integrity of normal cells, and demonstrates biocompatibility.

Apoptosis is a vital physiological process that restricts the growth of cell populations, either to preserve tissue homeostasis or to eliminate potentially hazardous cells, like those that have damaged DNA [70, 71]. The anticancer efficacy of STX against A549 was conveyed by its notable induction of apoptosis, as corroborated by CLSM (Fig. 11), annexin V-FITC apoptosis assay (Fig. 12), and the cell cycle arrest analysis (Fig. 13). Moreover, the apoptotic activity of STX against A549 is conferred by the activation of caspase-3, which induces cell death through apoptosis, as verified by real-time PCR analysis (Fig. 14A). Apocarotenoids significantly prompted alterations in cell morphology, nuclear condensation, DNA fragmentation, and mitochondrial dysfunction via the reduction of glutathione activity [72]. Additionally, they stimulated reactive oxygen species (ROS) generation, consequently triggering the activities of caspase 9 and caspase 3 [72, 73]. The preventive anticancer effects of carotenoids are primarily linked to their strong antioxidant properties, which can reduce ROS-induced DNA damage [74]. However, carotenoids act as pro-oxidant agents under conditions characterized by an unbalanced cellular redox state, high concentrations, and high oxygen tension. The pro-oxidative characteristics of carotenoids contribute to the generation of ROS in cancer cells, resulting in oxidative stress that limits cancer progression and metastasis. Pro-oxidants improve apoptosis by enhancing ROS-signaling pathways and/or compromising the antioxidant defenses of cancer cells [75]. Numerous studies have shown the pro-apoptotic effect of carotenoids through their antioxidant and pro-oxidant activities [75, 76]. Multiple research efforts have demonstrated that microbial pigments trigger apoptosis and impede the cell cycle of cancer cells [77, 78]. Mathew and Ramamoorthy, also reported the effect of apocarotenoids on breast adenocarcinoma MCF-7 cells through activation of apoptosis [79]. However, to date, there has been no reported study focusing on the role of STX as an agent targeting apoptosis.

In the recent decades, EGFR-targeted therapies have been recognized as the master orchestrator in regulating epithelial transformation and the behavior of cancer cells [80–82]. To the best of our knowledge, this is the first time in this study to report that the extracted STX pigment mitigates the expression of EGFR in cancer cells. The EGFR mutation creates a structural impediment for first- and second-generation EGFR-TKIs, leading to resistance by altering the crystal structure of the ATP binding pocket, hampering binding to the ATP binding site [83, 84]. The docking results indicated that the purified STX exhibited various bonding types with the mutated amino acids in the binding site via hydrophobic interactions and hydrogen bonding with additional amino acids. In light of the docking studies, STX pigment demonstrated a significant enzymatic binding efficacy with the wild and mutant EGFRs. However, further biochemical evidence was required to address the effectiveness of STX in combating the resistance associated with first- and second-generation EGFR-TKIs.

In conclusion, this research successfully enhanced the STX yields using BHI-cultivated *S. aureus* by nearly twofold and optimized cultivation methods for high-density pigment production. The application of RSM statistical design facilitated the multivariable optimization process, leading to an enhancement in the pigment amounts. Encouragingly, remarkable normal Vero cell tolerability has expanded STX prospective utility, which will open new avenues in the application of STX in the treatment of human diseases. It is worth mentioning that the biological finding reflects a pioneering effort in demonstrating an anticancer potential of STX, evidenced by its capacity in inducing apoptosis and mitigating EGFR expression in NSC lung cancer. Docking results obviously reveal that STX pigment could be considered a promising pioneer agent for further optimization as an EGFR inhibitor.

As a future perspective, more optimization trials could be screened using other possible factors, including salt concentration as another stress factor and mutagenesis for an enhanced STX synthetic pathway as an approach for maximized STX production. Moreover, additional in vivo validation, toxicity studies in animal models, or clinical trials are required to substantiate the safety profile and the efficacy of STX as a promising anticancer agent, evidenced by in vitro assays and molecular docking simulations.

## Data Availability

No datasets were generated or analysed during the current study.
